# Inhibition of MYC by the SMARCB1 tumor suppressor

**DOI:** 10.1038/s41467-019-10022-5

**Published:** 2019-05-01

**Authors:** April M. Weissmiller, Jing Wang, Shelly L. Lorey, Gregory C. Howard, Ernest Martinez, Qi Liu, William P. Tansey

**Affiliations:** 1 Department of Cell and Developmental Biology, Vanderbilt University School of Medicine, Nashville, TN 37232 USA; 2 Center for Quantitative Sciences, Vanderbilt University School of Medicine, Nashville, TN 37232 USA; 3 Department of Biochemistry, University of California at Riverside, Riverside, CA 92521 USA

**Keywords:** Cancer, Tumour-suppressor proteins

## Abstract

*SMARCB1* encodes the SNF5 subunit of the SWI/SNF chromatin remodeler. SNF5 also interacts with the oncoprotein transcription factor MYC and is proposed to stimulate MYC activity. The concept that SNF5 is a coactivator for MYC, however, is at odds with its role as a tumor-suppressor, and with observations that loss of SNF5 leads to activation of MYC target genes. Here, we reexamine the relationship between MYC and SNF5 using biochemical and genome-wide approaches. We show that SNF5 inhibits the DNA-binding ability of MYC and impedes target gene recognition by MYC in cells. We further show that MYC regulation by SNF5 is separable from its role in chromatin remodeling, and that reintroduction of SNF5 into *SMARCB1*-null cells mimics the primary transcriptional effects of MYC inhibition. These observations reveal that SNF5 antagonizes MYC and provide a mechanism to explain how loss of SNF5 can drive malignancy.

## Abstract (web summary)

SMARCB1/SNF5 is a tumour suppressor and component of the SWI/SNF chromatin remodeler. Here the authors show that, independent of chromatin remodelling activities, SNF5 acts to inhibit the pro-tumorigenic transcriptional program of MYC via control of RNA polymerase pause release at MYC target genes.

## Introduction

SWI/SNF is a multi-subunit chromatin remodeling complex that is frequently mutated in cancer^1^. Roughly 20% of all human cancers carry mutations in a SWI/SNF component^2^, a frequency approaching that of the tumor-suppressor *TP53*^1^. Although some tumor-associated alterations to SWI/SNF are gain of function and oncogenic^3^, the majority of mutations are loss of function^4^, pointing to a predominantly tumor-suppressive role for SWI/SNF complex members.

Perhaps the clearest example of a tumor-suppressive SWI/SNF protein is SNF5, a core component of the complex that is encoded by the *SMARCB1* gene (also known as *INI1* or *BAF47*)^5^. *SMARCB1* is a bona-fide tumor suppressor^6,7^ that is lost or inactivated in multiple malignancies, including malignant rhabdoid tumor (MRT)^8–10^, which is an aggressive and often lethal pediatric cancer. Interestingly, loss or inactivation of *SMARCB1* is the only recurring mutation in MRT—and often the only mutation detected in MRT genomes^11^—pointing to expansive functions of SNF5 in tumor suppression. Loss of SNF5 in MRT compromises SWI/SNF integrity, causing widespread collapse of enhancers regulating differentiation, and mobilization of residual SWI/SNF complexes to super-enhancers essential for tumor cell maintenance^12^. Conversely, reintroduction of wild-type SNF5 into MRT cell lines induces cell cycle arrest, apoptosis, purging of aneuploid cells, and loss of tumorigenicity^13–18^, demonstrating that the absence of SNF5 remains a driving force in the malignant state of these cells. It is possible that the tumor-suppressive actions of SNF5 are exerted entirely through its role in chromatin remodeling, but given the breadth of impact of SNF5 on cancer-relevant processes, it is equally possible that SNF5 plays a multi-faceted role in suppressing tumorigenesis.

In addition to functions within the SWI/SNF complex, SNF5 also binds to c-MYC^19–21^, an oncoprotein transcription factor with an extensive suite of protumorigenic activities^22^. SNF5 interacts directly with the carboxy-terminus of MYC^19,21^ and is proposed to stimulate the ability of MYC to transactivate its target genes^19^. The concept that SNF5 is a coactivator for MYC, however, conflicts with its well-established role as a tumor suppressor, with a report that SNF5 and MYC oppositely regulate a common set of genes^21^, with findings that loss of SNF5 in cancer is associated with activation of MYC target gene signatures^8–10^, and with recent observations that MYC inhibition can restrict rhabdoid tumor growth in vivo^23^. Given these disparities, it is clear that both the functional significance of the SNF5–MYC interaction—and the underlying mechanisms involved—are unresolved.

Here, we use a combination of biochemical and genomic approaches to interrogate how SNF5 impacts MYC. We demonstrate that SNF5 selectively inhibits the ability of MYC to bind DNA in vitro and in cells, and show that reintroduction of SNF5 into MRT cells results in a broad and comprehensive displacement of MYC from chromatin. By comparing SNF5 reintroduction with MYC inhibition, we further demonstrate that the actions of SNF5 on MYC are independent of its effects on chromatin remodeling, and instead are mediated via control of RNA-polymerase pause release at MYC-regulated genes. These observations show that SNF5 tempers target gene recognition by MYC, providing a mechanism to account for enhanced MYC function in MRT and suggesting that the tumor-suppressive functions of SNF5 are mediated, at least in part, by inhibiting MYC.

## Results

### SNF5 inhibits DNA binding by MYC

The carboxy-terminal basic helix-loop-helix leucine zipper (bHLHZip) region of MYC interacts with MAX to form a DNA-binding module that recognizes E-box DNA sequences (CACGTG)^22^. SNF5 binds within the bHLHZip, and although it has little if any effect on the MYC–MAX interaction^21^, the impact of SNF5 on the DNA-binding ability of full-length MYC:MAX heterodimers has not been determined.

**Fig. 1:**
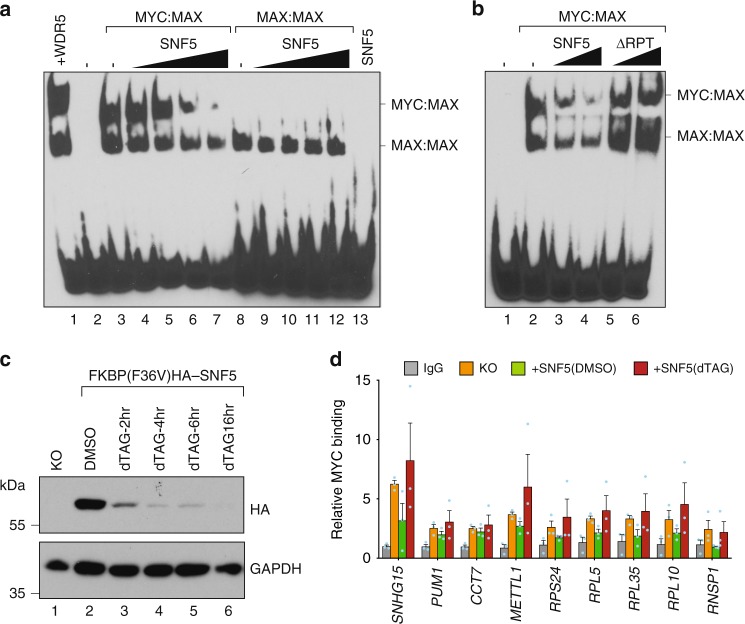
SNF5 inhibits DNA-binding by MYC. **a** Recombinant MYC:MAX or MAX:MAX complexes were incubated with increasing amounts of recombinant SNF5 (3-, 6-, 12-, 24-fold molar excess over MYC:MAX) and EMSA performed using a DNA probe carrying a wild-type E-box sequence. Note that MYC:MAX complexes also contain residual MAX:MAX dimers. Lane 1 shows that incubation with the MYC interactor WDR5 (12-fold molar excess) induces a supershift in the MYC:MAX complexes. Lane 13 is the top concentration of SNF5, without MYC or MAX proteins. **b** EMSA, as in **a**, except MYC:MAX complexes were incubated with either recombinant SNF5 (12-, 24-fold molar excess) or an SNF5 mutant lacking the imperfect repeats region (amino acids 176–309) that interact with MYC (∆RPT; 12-, 24-fold molar excess). Results were confirmed with two independent preparations of recombinant proteins. **c** *SMARCB1*-null (KO) HEK293 cells were transduced to express FKBP12^(F36V)−^HA-SNF5, treated with DMSO or 500 nM of dTAG-47 for the indicated times, lysates prepared, and the levels of HA-tagged degradable SNF5 determined by immunoblotting. Lysate from the KO cells is included as a control in lane 1. GAPDH is a loading control. **d** *SMARCB1*-null (KO) HEK293 cells were transduced to express FKBP12^(F36V)−^HA-SNF5 (+SNF5) and treated with DMSO or dTAG-47 (500 nM) for 2 h. ChIP assays were then performed. IgG control is shown for the dTAG-47-treated sample. Anti-MYC ChIPs are shown for the KO and the treated +SNF5 cells. Relative MYC binding is calculated as the signal at each of the indicated loci, relative to a non-MYC-bound locus (β-globin). *n* *=* 3 independent ChIP experiments. Error bars are standard error

First, we asked if SNF5 modulates DNA binding by MYC:MAX complexes in vitro. We reconstituted full-length MYC:MAX and MAX:MAX dimers from highly purified recombinant proteins^24^ (Supplementary Fig. 1a) and showed they specifically bind to E-box-containing DNA in an electrophoretic mobility shift assay (EMSA; Supplementary Fig. 1b). We added recombinant SNF5 (Supplementary Fig. 1a) to these reactions, and observed that increasing amounts of SNF5 resulted in displacement of MYC:MAX complexes from DNA (Fig. 1a, compare lane 3 with lanes 4–7). This effect was specific to MYC:MAX complexes, as contaminating MAX:MAX dimers in these preparations were less sensitive to SNF5 addition, and purified MAX:MAX complexes were refractory to the effects of SNF5 (lanes 8–12). The impact of SNF5 in these assays was not a general result of binding to MYC, as addition of the MYC-interaction partner WDR5^25^ did not disrupt DNA binding, but instead super-shifted MYC:MAX:DNA complexes (lane 1). Importantly, deletion of the conserved region of SNF5 containing two imperfect repeats—which mediate binding to MYC^19^—blocked SNF5-dependent displacement of MYC:MAX complexes from DNA (Fig. 1b, compare lanes 3 and 4 with lanes 5 and 6), showing that the ability of SNF5 to interact with MYC is required for disruption of DNA-binding. We conclude that SNF5 directly and selectively inhibits the DNA-binding ability of MYC in vitro.

Next, we asked if SNF5 modulates DNA binding by MYC in cells. We developed a system that allowed us to acutely manipulate SNF5 protein levels via the dTAG approach^26^. We knocked-out (KO) *SMARCB1* via CRISPR-mediated genome editing in HEK293 cells, and then expressed a form of SNF5 that is tagged with FKBP12^(F36V)^ (Supplementary Fig. 1c, d). In the presence of degrader dTAG-47 ^27^, tagged SNF5 was destroyed rapidly (Fig. 1c). Indeed, 2 h of treatment with dTAG-47 was sufficient to deplete most SNF5, with little if any effect on MYC protein levels (Supplementary Fig. 1e). We then used chromatin immunoprecipitation (ChIP) to monitor MYC binding to nine MYC-bound loci^28^ under each condition. Here, we found that re-expression of SNF5 in KO cells reduced MYC binding to chromatin at most of the genes tested (with the exception of *CCT7*), and that this effect could be rapidly reversed by dTAG-47-triggered degradation of SNF5 (Fig. 1d). Thus as we observed in vitro, SNF5 expression in cells can interfere with the ability of MYC to bind target genes in the context of chromatin.

### SNF5 inhibits target gene recognition by MYC in MRT cells

To look at the impact of SNF5 on MYC in a more tumor-relevant setting, we asked whether SNF5 modulates the interaction of MYC with chromatin in the context of MRT, which is driven by SNF5 loss, and where MYC target gene signatures are repeatedly activated. To determine if MYC is important in MRT cells, we first attenuated its expression and asked how this alters MRT cell viability and anchorage-independent growth. Here, we observed that shRNA-mediated knockdown of MYC significantly decreased both parameters (Supplementary Fig. 2), confirming the importance of MYC to MRT cells and reinforcing the notion that this is an appropriate setting in which to interrogate the influence of SNF5 on MYC.

We next established a system that allowed us to compare the effects of reintroduction of SNF5 in MRT cells with inhibition of MYC in the same setting. G401, an extensively studied (and MYC-dependent; Supplementary Fig. 2) MRT cell line^13–15,17,18^, was engineered to express inducible forms of enhanced green fluorescent protein (EGFP), SNF5, or OmoMYC—a dominant-negative mutant that blocks the productive association of MYC with its target genes^29–32^. In this experimental system the level of reintroduced SNF5 was comparable to endogenous SNF5 in other cell lines (Supplementary Fig. 3a). Reintroduced SNF5 interacted with the core SWI/SNF component BAF155 (Supplementary Fig. 3b), and co-migrated with other SWI/SNF complex subunit members in glycerol sedimentation assays (Supplementary Fig. 3c), consistent with its assembly into an SWI/SNF complex^12^. SNF5 also suppressed anchorage-independent growth of G401 cells in culture (Supplementary Fig. 3d, e). OmoMYC, as expected, reduced interaction of MYC with chromatin (Supplementary Fig. 3f). Notably, expression of SNF5 did not alter steady-state MYC protein levels (Supplementary Fig. 3g), providing the opportunity to look specifically at the effects of SNF5 on MYC function.

**Fig. 2:**
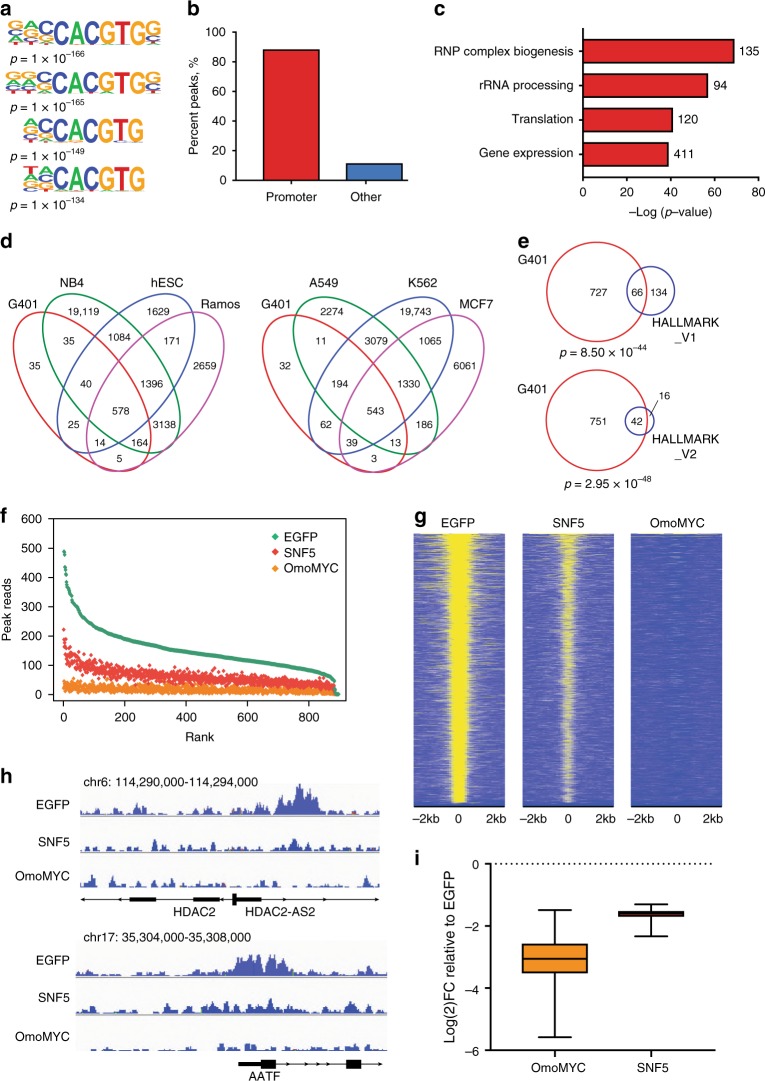
SNF5 inhibits chromatin binding by MYC in MRT cells. **a** Known motif enrichment analysis was performed on the ChIP-Seq data from G401 cells expressing EGFP. The top four motifs are shown; all are enriched in the E-box sequence (CACGTG). **b** MYC peaks in the EGFP-expressing cells were quantified in terms of their distance to the nearest annotated transcriptional start-site (TSS). Peaks within 1 kb of a TSS are called as “promoter” peaks. **c** MYC peaks located within 1 kb from a TSS were assigned to their nearest gene and GO term enrichment analysis was performed. **d** Overlap of MYC peaks obtained from EGFP-expressing G401 cells with six published MYC ChIP-seq data sets: NB4 acute promyelocytic leukemia cells (GSM935643), hESC human embryonic stem cells (GSM935509), Ramos Burkitt’s lymphoma cells (GSM762711), A549 lung cancer cells (GSM1003607), K562 chronic myeloid leukemia cells (GSM935516), and MCF7 breast cancer cells (GSM1006866). **e** MYC peaks that are located within 1 kb from a TSS were assigned to their nearest gene and overlaid with two MSigDB Hallmark MYC target data sets. A hypergeometric test was performed; significance is displayed below the Venn diagram. **f** Scatterplot of normalized peak read counts for each condition (average of replicates), ranked based on EGFP peak read number. **g** Heat maps of MYC peak intensity for each condition representing the combined average of normalized peak intensity in 100-bp bins ± 2 kb around the center of peaks. Genes ranked based on EGFP. **h** Two example genome browser tracks from each condition. **i** Box-and-whisker plot of log_2_-fold changes of MYC peaks for OMOMYC or SNF5 samples (FDR < 0.05), relative to EGFP. Box extends from 25th to 75th percentile with median marked by the middle line, whiskers extend from minimum to maximum point. *n* *=* 2 independent ChIP-seq experiments

First, we coupled ChIP to next-generation sequencing (ChIP-Seq) to track the distribution of MYC across the genome of G401 cells. We identified ~900 peaks of MYC binding in G401 cells expressing the EGFP control (FDR of 0.01). This number of peaks was relatively low, compared to what has been reported for other cell types^33^, but the pattern of binding we observed was authentic for MYC. Binding sites were enriched in the E-box motif (Fig. 2a), predominantly promoter proximal (Fig. 2b), and enriched in genes linked to the well-established role of MYC in stimulating protein synthesis^22^ (Fig. 2c). Comparison with six published ChIP-Seq data sets revealed that more than half of the MYC binding sites we tracked in G401 cells are shared with the other six cell types (Fig. 2d), while a hypergeometric test showed significant overlap of our G401 data with the two MSigDB Hallmark MYC target gene collections (Fig. 2e), both of which contain different MYC target genes. Reducing the analysis FDR to a more relaxed value of 0.1 increased the number of MYC peaks to ~1500, but did not substantively change any of the relevant characteristics (Supplementary Fig. 4). We conclude that the number of MYC binding sites in G401 cells is comparatively low, but that those sites that are bound are strongly connected to the core functions of MYC.

We next compared EGFP with the effects of OmoMYC or SNF5 expression in G401 cells (Fig. 2f–i). Here, OmoMYC reduced detectable MYC binding genome wide, consistent with its known functions^29–32^. Importantly, SNF5 also reduced MYC binding. The effects of SNF5 on MYC were genome-wide and the average magnitude of reduction in binding intensity was about threefold (Fig. 2i). As described, steady-state levels of MYC are unaffected by SNF5 expression at this same time point (Supplementary Fig. 3g), ruling out the possibility that the reduction in binding we observed is due to a decrease in MYC protein. These results are consistent with the response of MYC to altered SNF5 levels in HEK293 cells, and with our in vitro DNA-binding experiments, and demonstrate that SNF5 tempers the ability of MYC to bind chromatin in MRT cells.

### Chromatin regulation by SNF5 is distinct from effects on MYC

**Fig. 3:**
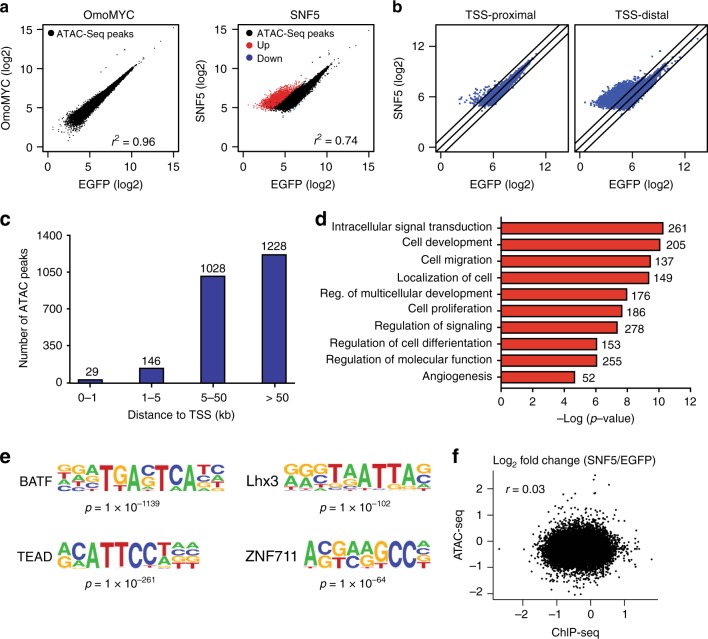
SNF5 causes changes in chromatin state that are independent of its impact on MYC. **a** Scatterplots showing log_2_-fold changes in ATAC-Seq peaks for OmoMYC and SNF5, compared to EGFP samples. **b** Differential ATAC-Seq peaks were separated based on whether they are TSS-proximal (≤1 kb upstream or 100 bp downstream of the TSS) or TSS-distal (greater than these parameters). **c** Graph presents a breakdown of the 2491 ATAC-Seq peaks induced by SNF5, according to their distance from the nearest annotated TSS. **d** GO analysis of genes associated with ATAC-seq peaks induced by SNF5 expression. **e** De novo motif analysis of ATAC-seq peaks induced by SNF5 expression, showing the top four motifs identified. **f** Scatterplot showing the log_2_-fold changes of MYC ChIP-Seq read counts (SNF5/EGFP) on the *x*-axis and the log_2_-fold changes of ATAC-Seq read counts (SNF5/EGFP) on the *y*-axis at all promoters

Given that reintroduction of SNF5 into MRT cells will reconstitute SWI/SNF^12^, it is possible that the effects we observe on MYC binding in our G401 system are due to alterations in chromatin accessibility triggered by the SWI/SNF complex. To ask whether SNF5 alters the chromatin landscape at or around MYC target genes, we used the assay for transposase accessible chromatin followed by next-generation sequencing (ATAC-Seq)^34^ to identify changes in chromatin accessibility induced by EGFP, SNF5, and OmoMYC expression. Overall, we identified ~25,000 sites of open chromatin in the EGFP-expressing cells. None of these sites were significantly affected by OmoMYC expression (Fig. 3a), indicating that displacement of MYC from its target genes in G401 cells does not substantively alter open chromatin status. In contrast, SNF5 expression resulted in a profound increase in chromatin accessibility, causing ~2500 new open chromatin sites to be formed (Fig. 3a). Only seven sites showed decreased accessibility upon SNF5 reintroduction (Fig. 3a). The majority of new open chromatin sites were transcription start site (TSS)-distal (Fig. 3b), with 90% being at least 5 kb from the nearest TSS, and half more than 50 kb away (Fig. 3c). Assignment of gained open chromatin peaks to their nearest gene showed a strong enrichment of genes involved in signal transduction, development, and differentiation (Fig. 3d). Moreover, gained sites are enriched for several DNA sequence motifs, including those belonging to the AP-1/ATF superfamily^35^ (Fig. 3e, Supplementary Table 1). These findings are consistent with the notion that SNF5 is important for enhancer regulation at critical cell identity genes^12,36,37^, and with published reports linking SNF5 to the AP-1/ATF proteins^12,36,37^. Importantly, the open chromatin peaks induced by SNF5 expression in G401 cells were entirely separable from MYC; there was no correlation between fold changes of MYC ChIP-Seq read counts (SNF5/EGFP) and ATAC-Seq read counts (SNF5/EGFP) at all promoters (Fig. 3f, *r* = 0.03), and no enrichment for E-box motifs in the ATAC-peak sequences (Supplementary Table 1). We conclude that reintroduction of SNF5 in G401 cells alters the chromatin landscape in a way that is consistent with its known functions, but that these effects are physically separable from its actions on MYC.

### SNF5 inhibits RNA polymerase pause release at MYC targets

A key transcriptional function of MYC is to modulate release of paused RNA polymerases at its target genes^38^. To determine if the ability of SNF5 to temper MYC binding to chromatin impacts this activity, we used PRO-Seq^39^, a global nuclear run-on approach, to compare how SNF5 and OmoMYC alter the distribution of active RNA polymerases in G401 cells, genome-wide and at near-nucleotide level resolution. PRO-Seq also allowed us to follow primary transcriptional effects, and at the same time point (24 h postinduction) as our ChIP- and ATAC-Seq experiments.

**Fig. 4:**
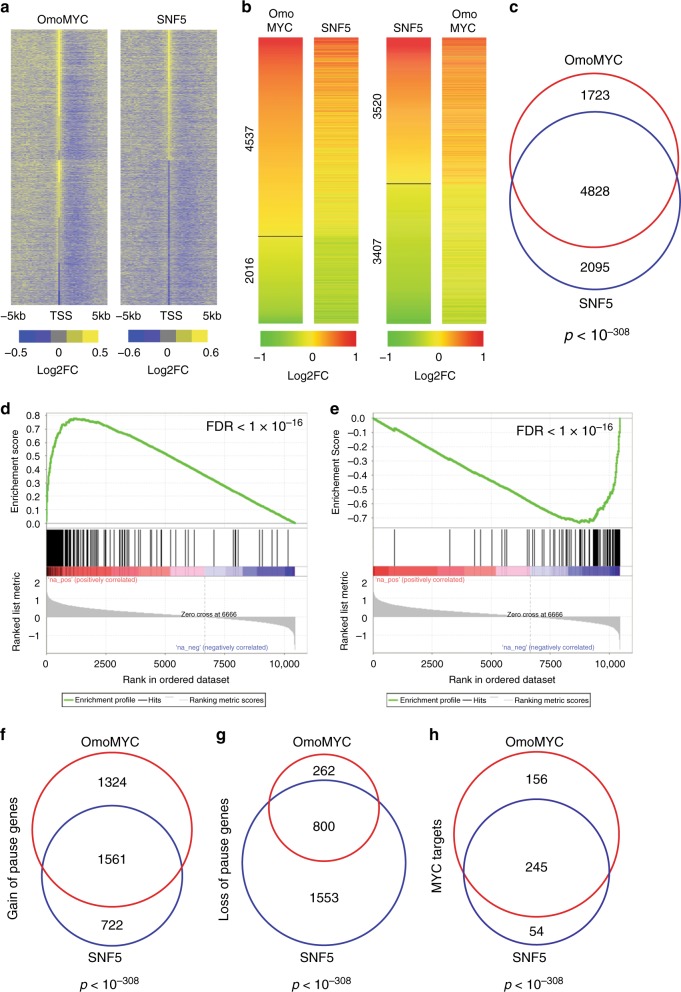
SNF5 mimics the primary transcriptional effects of MYC inhibition. **a** Heatmaps displaying log_2_-fold change (Log2FC) of active polymerases (compared to EGFP) in the promoter-proximal region and ±5Kb around the transcription start site (TSS), as determined by PRO-Seq. The two heatmaps are sorted in the same order; genes with increase in both OmoMYC and SNF5, genes with increase in OmoMYC only, and other active genes. Genes in each group are ranked by the OmoMYC FC near the TSS. **b** Heatmaps of genes showing changes in pausing index for OmoMYC and SNF5, compared to EGFP. Left panel: genes are ranked based on log_2_-fold change of pausing index in OmoMYC, and the corresponding changes in SNF5 are shown on the right side. Right panel: genes are ranked based on log_2_-fold change of pausing index in SNF5, and the corresponding changes in OmoMYC are shown on the right side. **c** Overlap of unique genes that had a change in pausing index upon OmoMYC expression with those that had a change following expression of SNF5 (FDR < 0.05). **d** Gene set enrichment analysis showing the distribution of genes that had an induced pause following SNF5 reintroduction against the ranked list of all genes with change in pausing index following OmoMYC expression (FDR < 0.05, genes ranked by log_2_-fold change). **e** Gene set enrichment analysis, as in **d**, except for genes that had loss of pause following SNF5 reintroduction. **f** Overlap of genes that had increased pausing index (induced pause) for both OmoMYC and SNF5 (FDR < 0.0001). **g** Overlap of genes that had decreased pausing index (pause release) for both OmoMYC and SNF5 (FDR < 0.0001). **h** Overlap between the number of genes identified as MYC targets from ChIP-seq analysis (Fig. 3) that also had a resulting induced pause for each condition. *n* *=* 3 independent PRO-Seq experiments

Compared to the EGFP control, OmoMYC and SNF5 induced a large number of transcriptional changes in the distribution of active RNA polymerases, both proximal to promoters and further inside gene bodies (Fig. 4a). Consistent with the role of MYC in promoting RNA polymerase pause release, OmoMYC increased the pausing index—the ratio of active polymerases at the promoter versus the gene body^40^—at ~4500 genes (Fig. 4b), with a smaller number (~2000) of genes showing a decrease in this ratio. SNF5, in contrast, produced an almost equal number of increases (~3500) and decreases (~3400) in pausing index. Notably, when we compared these two data sets, we found that ~70% of the genes that show a change in pausing index with SNF5 are also changed with OmoMYC (Fig. 4c). Separating these genes according to the direction of change, we observed a highly significant correlation between the extent to which polymerase pausing was altered by OmoMYC and SNF5, both for genes showing a gain (Fig. 4d), as well as a loss (Fig. 4e), of pause. In total, ~70% of genes gaining a pause with SNF5 also gained a pause with OmoMYC (Fig. 4f), and ~35% of genes losing a pause with SNF5 lost a pause with OmoMYC (Fig. 4g). Principal component analysis of pausing indices revealed that the transcriptional effects of OmoMYC and SNF5 cluster more closely, and therefore have a similar effect, at MYC-bound, compared to MYC-unbound, genes (Supplementary Fig. 5a). And quantitative comparison of pausing index differences indicated that changes in pausing index were similar between OmoMYC and SNF5 at MYC-bound genes, but different at MYC-unbound loci (Supplementary Fig. 5b). Eighty percent of the MYC targets that gain a pause with SNF5 gain a pause with OmoMYC (Fig. 4h), a significantly higher level of overlap than for non-MYC targets (Supplementary Fig. 6a). In contrast, only 35% of MYC target genes experience a loss of pause under both conditions (Supplementary Fig. 6b). In general, the extent and significance of overlap between SNF5 and OmoMYC was higher for genes experiencing RNA polymerase pause induction, and the types of genes regulated in each direction were different (Supplementary Fig. 6c, d), with pause-induced genes being enriched in those connected to canonical MYC functions, including protein synthesis. Based on these data, we conclude that reintroduction of SNF5 in G401 cells mimics many of the transcriptional effects of MYC inhibition, and that a major impact of SNF5 on transcriptional events is to promote pausing of RNA polymerase at genes regulated by MYC.

Finally, we asked if these changes in RNA polymerase distribution correlate with the known effects of SNF5 reintroduction on the transcriptome of G401 cells. Comparing our PRO-Seq data to published RNA-Sequencing (RNA-Seq) data sets gathered at 3 or 7 days after reconstitution of G401 cells with SNF5^37^, we observed highly significant correlations between the two: At day 3, ~30% of the genes that gain a pause with both SNF5 and OmoMYC in our experiments showed reduced RNA levels by RNA-Seq (Supplementary Fig. 6e), and at day 7 this overlap was 40% (Supplementary Fig. 6f). In contrast, there was no significant overlap between genes that lose a pause and those showing an increase in RNA levels at either time point (Supplementary Fig. 6g, h). The correlation between our PRO-Seq data and these published RNA-Seq data strongly implies that the ability of SNF5 to induce RNA polymerase pausing at genes regulated by MYC represents a significant mechanism through which it shapes the transcriptome.

## Discussion

Given the frequency with which SWI/SNF components are perturbed in malignancy^2^, understanding the mechanisms through which alterations in SWI/SNF drive tumorigenesis is fundamental to understanding how many cancers form and how they can be treated. Among SWI/SNF-mutant cancers, those defined by loss of SNF5 are particularly intriguing. On one hand, these cancers have an unusually simple genetic profile, with a single driver mutation—loss of *SMARCB1*/SNF5—and little if any evidence of collaborating oncogenic events. On the other hand^11^, these cancers are early onset malignancies^41^ that are difficult to treat and most often lethal. The contrast between the genetic simplicity of cancers like MRT and their aggressive nature implies that loss of SNF5 leads to a multitude of pro-oncogenic effects. Here, we provide evidence that one part of tumor suppression by SNF5 is to temper MYC binding to DNA. The direct connection between SNF5 and MYC explains the recurring activation of MYC target gene signatures in MRT^8–10^ and, because of the broad suite of oncogenic activities possessed by MYC^22^, can help rationalize how loss of a single tumor suppressor can have such profound effects on cellular pathophysiology.

The evidence that SNF5 directly impedes DNA binding by MYC is compelling (Fig. 1), and in line with recent NMR-based studies showing that the imperfect repeats of SNF5—which are required for this activity (Fig. 1b)—recognize the DNA-binding surface of the MYC:MAX bHLHZip heterodimer in a manner that is mutually exclusive with DNA recognition^42^. What we do not know, however, is the biochemical context in which SNF5 tempers MYC in cells. We see (Supplementary Fig. 3c), as others have reported^12,37^, that SNF5 that is reintroduced into MRT cells is incorporated into an intact SWI/SNF complex, suggesting that there is little unincorporated SNF5 in our experiments. We also see in these experiments changes in chromatin accessibility (Fig. 3) that are consistent with functional reconstitution of SWI/SNF. We cannot, however, exclude the possibility that there is some low level of free SNF5 that inhibits MYC binding, or that SWI/SNF transiently donates SNF5 for MYC inhibition. Regardless of the context, however, the ability of SNF5 to interfere with target recognition by MYC in vitro and in two different cellular systems demonstrates a clear biochemical mechanism through which SNF5 antagonizes a key MYC activity.

**Fig. 5:**
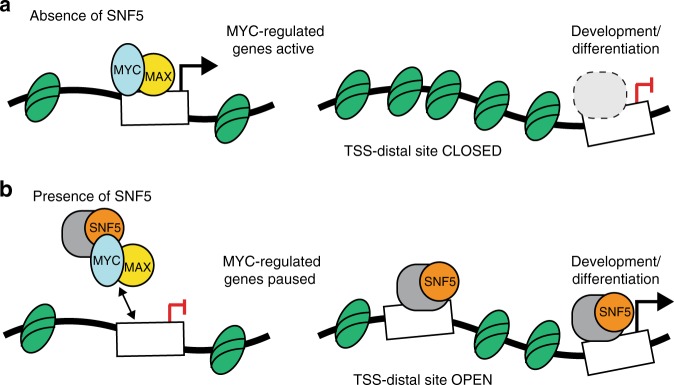
Model: Dual tumor-suppressor activities of SNF5. **a** In the absence of SNF5, MYC binds unimpeded to chromatin to promote gene expression programs that maintain the MRT state. Under these conditions, transcription start site (TSS)-distal sites that regulate transcription of genes involved in development and differentiation remain in a closed state, repressing transcription. **b** When SNF5 is present, MYC binding to DNA is tempered and RNA polymerase pauses at these genes, repressing transcription. SNF5, in concert with the SWI/SNF complex (gray), also leads to remodeling at TSS-distal sites, resulting in an open chromatin state and activation of genes linked to development and differentiation

The data presented here show that reintroduction of SNF5 into MRT cells leads to changes in TSS-distal chromatin accessibility at sites connected to differentiation and development (Fig. 3), very much in line with recently documented functions of SNF5 in maintaining lineage-specific enhancers and cell identity^12,36,37^ and activating bivalent promoters at developmentally important genes^37^. By comparing these activities with the location of MYC in G401 MRT cells, we show that SNF5 has a second set of activities—against MYC—that are almost exclusively promoter-proximal (Fig. 2), and induce RNA polymerase pause arrest at genes regulated by MYC (Fig. 4). Our ability to physically separate the canonical functions of SNF5 from its anti-MYC activities reveals that SNF5 does not modulate MYC binding via changes in chromatin accessibility, and supports a revised model in which dual regulation of both chromatin accessibility (at TSS-distal enhancers) and control of MYC (at TSS-proximal promoters) are part of the SNF5 tumor-suppression program (Fig. 5).

What is the functional significance of a dual tumor-suppression mechanism for SNF5? In cells such as G401, which possess a modest number of MYC binding sites, almost all of which are promoter-proximal (Fig. 2), these two mechanisms would impact distinct sites in the genome, allowing SNF5 to support both the broad transcriptional patterning achieved by enhancer/super-enhancer regulation, as well as the gene-specific control that can be achieved via targeting a sequence-specific transcription factor such as MYC. By directly modulating MYC at promoters, SNF5 would have the ability to couple enhancer-mediated cell identity determination with essential ancillary processes such as regulation of the cell cycle and biomass production (Supplementary Fig. 6c); an activity that would be critical during processes such as development—where SNF5 plays a key role in maintaining the balance between pluripotency and differentiation^43^—but upon SNF5 loss could readily conspire to drive tumorigenesis. Indeed, although the promoter-specific function of SNF5 in MRT has not previously been reported, our PRO-Seq studies clearly show that SNF5 impacts primary transcriptional events at MYC target genes in G401 cells and mimics MYC inhibition. Many of these changes in polymerase distribution correlate with changes in transcript levels, implying that the changes we see are relevant to disease processes in MRT. We also point out that the separable functions of SNF5 in regulating chromatin structure and tempering MYC do not necessarily have to operate at distinct regions of the genome. High levels of MYC overexpression lead to broad invasion of promoters as well as enhancers by MYC^44^, and are associated with MYC binding to degenerate E-box elements. In cells with a high MYC burden, therefore, MYC that is bound at enhancers may be particularly sensitive to inhibition, and the tumor-suppressive functions of SNF5 could act within the context of SWI/SNF to both maintain normal patterns of open chromatin status at key enhancers and to resist cooption of enhancer function by ectopic MYC.

One of the challenges in treating cancers such as MRT is that loss of a key tumor suppressor such as SNF5 does not readily reveal a strategy where drug-like molecules—most of which are inhibitors—could be effective. Most children diagnosed with MRT die quickly from highly metastatic disease, despite treatment regimens that can involve combinations of surgery, chemotherapy, and radiation. Some modest improvements in patient survival have been made in recent years^45^, but there is currently no standard of care for treating MRT sufferers, and the chances that a child diagnosed with MRT will survive a year, let alone 5 years, is very small^45^. Our data strongly imply that loss of SNF5 drives MRT, in part, by derepressing MYC. Although there are no drug-like MYC inhibitors available at present, the importance of MYC to human cancer fuels intense interest in their discovery, and a variety of direct and indirect methods to target MYC in the clinic are being moved forward^46^. Many of these approaches target the interaction of MYC with chromatin. OmoMYC itself is being developed in this capacity^46^, and has in vivo action against atypical teratoid rhabdoid tumors^23^, which (like MRT) are caused by SNF5 loss. The striking parallels we see between SNF5 reintroduction and OmoMYC expression in G401 cells—as well as our finding that MRT cell lines depend on MYC for viability—lays a strong conceptual foundation for the idea that MYC inhibition would be unexpectedly effective in treating this malignancy, and others driven through inactivation of SNF5.

## Methods

### Cell culture and transductions

G401, A204, and HEK293 cell lines were obtained from ATCC and maintained in Dulbecco's Modified Eagle Medium (DMEM) supplemented with 10% FBS and 1% penicillin/streptomycin, or RPMI with 10% fetal bovine serum (FBS) and 1% penicillin/streptomycin, respectively. HEK293 cells were maintained in DMEM supplemented with 10% FBS and 1% penicillin/streptomycin. All cell lines used were confirmed as mycoplasma-negative. For inducible constructs, lentiviral transductions were performed by transfecting HEK293 cells with the appropriate inducible construct, the pMD2.G envelope expressing plasmid, and the psPAX2 packaging plasmid, which were gifts from Didier Trono (Addgene plasmid #12259 and #12260, respectively). Viral supernatant was collected in DMEM with 10% TET-system approved FBS (Clontech 631106) and 1% penicillin/streptomycin, and used to infect G401 cells. Stable cells were selected for 6 days using 0.25 mg/ml G418 in DMEM with 10% TET-system-approved FBS and 1% penicillin/streptomycin. After selection, cells were counted, treated with 1 μg/ml doxycycline (Sigma D9891) for 24 h, and experiments performed as described below. For shRNA constructs, transductions were performed as described, but viral supernatant was collected in normal FBS maintenance media and used to infect G401 and A204 cells. Cells were transduced for 2 days with shRNA viral supernatants, allowed to recover 1 day, and then a portion of the cells were counted on day 3, and at each time point, by flow cytometry as described below. For dTag experiments, transductions were performed as described above with normal FBS maintenance media and those viral supernatants were used to infect an HEK293-SNF5-KO clone. Cells were transduced for 2 days and expanded for an additional 4–6 days before plating for ChIP experiments as described below.

### Generation of SMARCB1-knockout cell lines

HEK293 cells were transfected with the *Ini1* CRISPR/Cas9 KO Plasmid (Santa Cruz Biotechnology, sc-401485) and *Ini1* HDR Plasmid (Santa Cruz Biotechnology, sc-401485-HDR) using Lipofectamine 3000 (Life Technologies), following the manufacturer’s protocol. After 72 h, cells were plated into a large dish with puromycin to allow for individual colonies to form. Individual colonies representing single clones were amplified and screened for the presence of SNF5 using two independent antibodies to identify SNF5-null cell lines.

### Flow cytometry analysis

G401 and A204 cells transduced with appropriate shRNA vectors were collected and resuspended in DMEM with no phenol red. 2.5–5 × 10^5^ cells were filtered through a 35 µm nylon mesh Falcon round bottom test tube and the remaining cells returned to culture until the next time point. Filtered cells were delivered to the Vanderbilt Flow Cytometry Shared Resource for analysis of the number of GFP-positive cells using a Becton Dickinson LSRFortessa instrument. For each time point, 20,000 cells were counted using forward and side scatter to select single cells. The number of GFP-positive cells in the population was expressed as a percent of single, nonaggregated, cells. Initial experiments performed with addition of propidium iodide confirmed little to no toxicity of shRNA expression in GFP-positive cells. The gating strategy used for flow cytometry experiments is shown in Supplementary Fig. 7.

### Creation of lentiviral constructs

Lentiviral shRNA constructs were designed by VectorBuilder (Cyagen) to include either a scrambled shRNA or one of two shRNAs against human MYC shRNA (from their database), driven by the U6 promoter. Each construct also contained an EGFP:T2A:Puromycin cassette driven by the PGK promoter. All lentiviral shRNA constructs were validated in HEK293 cells after selection with puromycin to confirm knockdown of MYC. Tet-inducible lentiviral vector OMOMYC was created by introducing four mutations^29^ into the DNA-binding domain of MYC by site-directed mutagenesis. OMOMYC was then PCR amplified and inserted into the multiple cloning site of pENTR1A^47^, with sequences encoding an HA-epitope tag, via Gibson assembly. pENTR1A (no ccDB (w48–1)) plasmid was a gift from Eric Campeau & Paul Kaufman (Addgene plasmid #17398). EGFP was amplified from the pEGFP-N1 plasmid (Clontech) and SNF5 was amplified from the pFastBac1 INI1 construct, which was a gift from Robert Kingston (Addgene plasmid #1953); both were inserted into the pENTR1A plasmid with sequences encoding an HA-epitope tag. Gateway cloning was used to insert each pENTR1A fragment into the lentiviral pInducer20 acceptor vector^48^, which was a gift from Stephen Elledge (Addgene plasmid #44012). To create a degradable SNF5 construct, the pENTR1A fragment containing SNF5 was gateway cloned in frame into the lentiviral pLEX-305-N-dTAG, a gift from James Bradner (Addgene plasmid #91797)^26^. All plasmid constructions were confirmed by DNA sequencing.

### Electrophoretic mobility shift assays

SNF5 coding sequences were amplified from the pFASTBac1 INI1 construct and inserted into a pSUMO expression vector^25^, carrying an N-terminal 6×-HIS tag for purification. SUMO-WDR5^25^, SUMO-SNF5, SUMO-SNF5ΔRPT, and a control SUMO construct encoding amino acids 533–580 of BAF155, were expressed in Rosetta *E. coli* cells (EMD Millipore) and purified using a nondenaturing lysis buffer and Ni-NTA agarose, according to the Ni-NTA QIAGEN protocol (Qiagen). SUMO-SNF5ΔRPT was engineered by deleting amino acids 176–309 in SUMO-SNF5 using Q5 whole-plasmid mutagenesis (NEB). Recombinant 6×-HIS MYC and MAX were expressed in Rosetta *E. coli* cells and purified as previously published^24^. Functional MYC:MAX heterodimers were formed by combining a 3:1 molar ratio of MYC to MAX and performing step-wise dialysis^24^. DNA-binding reactions were performed using a double-stranded E box oligonucleotide bearing a 3′-biotin group: 5′-GCTCAGGGACCACGTGGTCGGGGATC-3′ (IDT). The mutant E box oligonucleotide two changes (5′-GCTCAGGGACCAGCTGGTCGGGGATC-3′). Binding reactions were performed in a 10–15 μl final volume and contained 15 mM Tris-HCl (pH 7.9), 15% glycerol, 100 mM KCl, 0.15 mM ethylenediaminetetraacetic acid (EDTA), 0.075% NP-40 (v/v), 7.5 mM 2-mercaptoethanol, 40 ng poly(dI-dC), 375 ng/μl BSA and 25 fmol biotin-E box probe. All binding reactions were performed for 30 min at room temperature with 0.01 pmol MAX:MAX or 0.4–0.8 pmol MYC:MAX per reaction. For competition experiments, unlabeled wild-type, or mutant E box oligos, were added in molar excess over the biotin-E box probe. For SNF5 experiments, SUMO-SNF5, SUMO-SNF5ΔRPT, and SUMO-WDR5 were added in molar excess over MYC:MAX or MAX:MAX and incubated for 30 min at room temperature before the biotin-E-box probe was added. To control for off-target effects of alterations in protein levels, a SUMO-fusion protein encoding amino acids 533–580 of BAF155 was added to maintain the same total molar ratio of protein in each binding reaction. Samples were resolved on a pre-run 5% native polyacrylamide gel in 0.5× TBE (45 mM Tris-borate, 1 mM EDTA) gel for 25 min at 150 V and electroblotted to a nylon membrane (GE Healthcare) for 30 min at 100 V in 0.5× TBE. The membrane was dried and cross-linked by ultraviolet light for 1 min using the optimal crosslink setting on a Spectroline UV Crosslinker Select Series. Detection and visualization of bands was accomplished using the LightShift Chemiluminescent EMSA kit (ThermoFisher Scientific) according to the manufacturer’s instructions.

### Glycerol sedimentation assay

G401 cells expressing inducible SNF5-HA were plated at 10 × 10^6^ per plate and treated with 1 μg/ml doxycycline for 24 h. Cells were collected and lysed by dounce homogenization in Buffer A (10 mM HEPES (4-(2-hydroxyethyl)-1-piperazineethanesulfonic acid), pH 7.6, 25 mM KCl, 1 mM EDTA, 10% glycerol, 1 mM dithiothreitol (DTT) with Protease Inhibitor Cocktail; Roche). Nuclei were pelleted at 500 × *g* for 5 min, resuspended in Buffer B (10 mM HEPES, pH 7.6, 3.5 mM MgCl_2_, 100 mM KCl, 0.1 mM EDTA, 10% glycerol, 1 mM DTT with Protease Inhibitor Cocktail; Roche), and then lysed by addition of ammonium sulfate at a final concentration of 0.3 M. Insoluble chromatin fraction was removed by centrifugation (100,000 × *g*) for 20 min in a TLA 100.3 rotor using a tabletop ultracentrifuge (Beckman). To the soluble fraction, 0.3 mg/ml ammonium sulfate was added and allowed to incubate for 20 min on ice. Precipitated proteins were recovered by centrifugation (100,000 × *g*) for 30 min in a TLA 100.3 rotor. Precipitated proteins were resuspended in an HEMG buffer containing no glycerol (25 mM HEPES, pH 7.9, 0.1 mM EDTA, 12.5 mM MgCl_2_, 100 mM KCl, 1 mM DTT) and then overlaid onto a 10-ml 10–30% glycerol gradient (in HEMG buffer). Tubes were centrifuged at 4 °C for 18 h at 283,000 × *g*. Fractions (0.5 ml) were collected and used to probe proteins by western blot analysis.

### Anchorage-independent growth assay

After transduction and selection were complete, 2000 G401 cells expressing TET-inducible EGFP, or SNF5, were mixed with 0.4% agarose-supplemented DMEM (with TET-approved FBS and 1% penicillin/streptomycin with 1 μg/ml doxycycline) and added slowly onto a solidified 0.8% agarose bottom layer. Fresh 1 μg/ml doxycycline was added every 2–3 days for a total of 14 days. Cells were fixed and stained with 0.05% crystal violet in 70% methanol overnight at room temperature and then destained with extensive washing with water. All plates were randomly labeled and analyzed blindly. For A204 cells, the same method was used, except media was replaced once a week for 28 days.

### Western blotting and antibodies

G401 cells induced with 1 μg/ml doxycycline for 24 h (and other cells used in comparing SNF5 levels) were collected in a lysis buffer (150 mM Tris-HCl pH 8.0, 150 mM NaCl, 5 mM EDTA, 1% Triton X-100 with Protease Inhibitor Cocktail; Roche), sonicated at 25% power for 15 s, and cleared by centrifugation. Protein concentrations were quantified using the Bio-Rad Bradford assay and 10 μg of lysate resolved by SDS-PAGE. For HA-coimmunoprecipitation experiments, nuclei from induced cells were extracted in 10 mM HEPES, pH 7.9, 10 mM KCl, 0.4% NP-40 and then lysed in lysis buffer. Equal amounts of nuclear lysates were subjected to immunoprecipitation with 5 µl of HA-tag antibody overnight at 4 °C, bound to protein A agarose (Roche), washed four times in lysis buffer, and resolved by SDS-PAGE. Resolved proteins were transferred to PVDF membrane (PerkinElmer) and blocked in 5% milk in TBS-T (50 mM Tris, pH 7.5, 150 mM NaCl, 0.1% Tween-20). Immunoblotting was performed using the following antibodies: SNF5 (Bethyl Laboratories, A301-087A, Abcam, ab12167, and Cell Signaling, 91735; all used at 1:1000 dilution), BAF155 (Cell Signaling, D7F8; 1:1000), GAPDH-HRP (Invitrogen, MA5-15738; 1:50,000), HA-epitope tag (Cell Signaling, C29F4; 1:1000), HA-HRP (Roche, 12013819001; 1:2000), MYC (Santa Cruz Biotechnology, sc-274; 1:500; Abcam, Y69; 1:500), and MAX (Santa Cruz Biotechnology, sc-275; 1:500). Visualization of bands were detected using Supersignal West Pico (Pierce).

### ChIP and ChIP-Seq

Transduced cells were plated at 10 × 10^6^ per plate and treated with 1 μg/ml doxycycline for 24 h. Cells were crosslinked using 1% formaldehyde for 10 min, quenched with 0.125 M glycine for 10 min, washed with ice-cold PBS two times, and collected by centrifugation. Nuclei were extracted in 10 mM HEPES, pH 7.9, 10 mM KCl, 0.4% NP-40 and then incubated in 1× TE (10 mM Tris, pH 8.0, 1 mM EDTA) with 1% SDS for 15 min on ice. Chromatin was fragmented using sonication with a Diagenode Biorupter, and debris was removed by centrifugation. Chromatin was frozen at −80 °C until ready to use. For dTag experiments, HEK293 cells expressing a degradable version of SNF5, cells were plated at 10 × 10^6^ 4–6 days post transduction. Then, 500 nM dtag-47 or matched DMSO control were added for 2 h and then the above protocol was used to harvest chromatin. Each immunoprecipitation was performed on chromatin collected from 10 × 10^6^ cells by dilution in ten volumes of FALB buffer (50 mM HEPES, pH 7.5, 140 mM NaCl, 1 mM EDTA, 1% Triton) using antibodies against MYC (N262, Santa Cruz Biotechnology, sc-764 or Cell Signaling, 9402; 3 µg) or normal rabbit IgG control (Cell Signaling, 2729 s; 3 µg). Immunoprecipitated DNA was bound to protein A agarose (Roche) and washed sequentially with low salt buffer (20 mM Tris, pH 8.0, 150 mM NaCl, 2 mM EDTA, 1% Triton), high salt buffer (20 mM Tris, pH 8.0, 500 mM NaCl, 2 mM EDTA, 1% Triton), Lithium Chloride Buffer (10 mM Tris, pH 8.0, 25 mM LiCl, 1 mM EDTA, 1% Triton) and two times with 1× TE. Agarose beads were then resuspended in 1× TE + 0.1% SDS + 20 µg proteinase K and incubated overnight at 65 °C. For ChIP, samples were diluted in 1× TE and coprecipitating DNAs quantified by Q-PCR using the following primers: SNHG15 (CGCCACTGAACCCAATCC and TCTAGTCATCCACCGCCATC), PUM1 (TATGAAGGGACAATCTGCTC and AATCCATCTTCATCCTACCG), CCT7 (TTCCAAAATGATGGTGAGTG and AGAGGGTCCTACAGAGCAAG), METTL1 (GCATGGCTGCGTCATTAACT and GAGTCTCGGCTGCCATGAT), RPS24 (TTGGCTGTCTGAAGATAGATCG and CGCGTGCCTATAGCTCAAGT), RPL5 (CCTGCAGGTCTCTGTCGAG and GGCATACGGGCAAGAAAAG), RPL35 (CTTGTGCAGCAATGGTGAGA and GCCTAGGTGGCAGATAGAATC), RPL10 (GCAAGAGTTCTACGCCCAAG and CACATGCGCAGATCAGAGAG), RNSP1 (GATGTAAGTTGGGGCGGAAT and GAGGAGTGGACCGGCTTC), and SNHG15 GB (AATTATGTGTCCAGGGTTGC and CACCGGCTTCTATATTCCAC). For ChIP-Seq, DNA from the equivalent of 30 × 10^6^ cells was combined and purified using a Qiagen PCR purification kit, and eluted DNA was used to generate libraries. Libraries were made using NEBnext Ultra II DNA library Prep protocol and NEBNext Multiplex Oligos for Illumina, with the addition of an AMPure clean-up step prior to beginning end repair. Sequencing data were obtained on an Illumina NextSeq500 with 75 bp single reads. Sequencing was performed by the VANTAGE Core at Vanderbilt University.

### ATAC-Seq

Cells were plated at 1 × 10^6^ per plate with 1 μg/ml doxycycline for 24 h and then 75,000 cells were harvested following published protocols^34,49^. Briefly, cells were harvested and nuclei were extracted in 10 mM Tris-HCl, pH 7.4, 10 mM NaCl, 3 mM MgCl_2_, 0.1% IGEPAL CA-630 (Sigma). Nuclei were immediately added to the transposase reaction containing Tn5 Transposase (Illumina Nextera DNA Kit) for 30 min at 37 °C. Reactions were stopped by purification with the Qiagen PCR Purification Kit. Transposed DNA was used to generate libraries through PCR amplification with NEBNext High-Fidelity 2× PCR Master Mix and Nextera-based primers (obtained from IDT). Amplified libraries were submitted to Genewiz for sequencing on an Illumina HiSeq2500 with 50 bp paired end reads.

### PRO-Seq

Cells were plated at 10 × 10^6^ per plate with 1 μg/ml doxycycline for 24 h and then 30 × 10^6^ cells were combined per condition and harvested together according to published protocols^50^, with minor changes. Briefly, nuclei were extracted in 10 mM Tris-HCl, pH 7.4, 300 mM sucrose, 10 mM KCl, 5 mM MgCl_2_, 1 mM EGTA, 0.05% Tween, 0.1% NP-40, 0.5 mM DTT, RNAse inhibitor and protease inhibitor cocktail (Roche) and then snap-frozen in 10 mM Tris-HCl, pH 8.0, 25% glycerol, 5 mM MgCl_2_, 0.1 mM EDTA, 5 mM DTT with protease inhibitor cocktail and stored at −80 °C until ready to use. Biotin run-on reactions were performed on thawed nuclei in a reaction buffer containing Biotin-11-CTP (PerkinElmer, NEL542001) for 3 min at 30 °C. Reactions were stopped by adding Trizol LS (Thermo Scientific) and RNA purified by chloroform and isopropanol extraction. Resuspended RNA pellets were heated at 65 °C for 40 s and 1 M NaOH was added, followed by incubation on ice for 10 min. Base hydrolysis was neutralized by addition of 1 M Tris (pH 6.8) and the sample ran over a Micro Bio-Spin P-30 gel column (Bio-Rad). Strepavidin Dynabeads (ThermoFisher, 65601) were incubated with collected material and biotinylated RNA bound to beads using their standard protocol. Elution of bound, biotinylated, RNA was achieved by extracting beads with Trizol and subsequently purified using chloroform and isopropanol. RNA adaptors (IDT) were added to the 3′ side biotinylated RNA and following a second round of biotin-RNA purification, 5′ RNA caps were removed using CAP CLIP (CellScript, C-CC15011H). 5′ RNA adaptors (IDT) were then added. One additional biotin-RNA purification was performed, and purified RNA was used in a reverse transcriptase reaction to generate cDNA. Libraries were amplified using the generated cDNA and a PCR cycle number determined from a test analysis of a portion of sample. Library amplification was performed with Phusion high-fidelity polymerase (*NEB*) and customized Illumina-based index primers (*IDT*). Sequencing data were obtained on an Illumina NextSeq500 with 75 bp single reads. Sequencing was performed by the VANTAGE Core at Vanderbilt University.

### ChIP-Seq processing and analysis

ChIP-seq reads were aligned to the human genome using Bowtie2^51^. Peaks in each sample were called using MACS2 with *q* value of 0.01^52^. Peaks were annotated using Homer command annotatePeaks, and enriched motifs were identified by Homer command findMotifsGenome with the default region size and the motif length (-size 200 and -len 8, 10, 12) (http://homer.ucsd.edu/homer/). Consensus peaks in each condition were identified using DiffBind^53^, where peaks occurring both replicates were included. Peaks identified across conditions were combined into a final peak set and ChIP read counts were calculated for the final peak set. Read counts were normalized to the total mapped reads, and differential peaks were determined by DESeq2^54^, which calculated the log_2_ fold changes, Wald test *p* values, and adjusted *p* values (False Discovery Rate, FDR) by the Benjamini–Hochberg procedure. The significantly changed peaks were assessed with an FDR < 0.05. Hypergeometric test was used to estimate the enrichment of MYC target genes in MSigDB Hallmark data sets using all human genes as background. The overlap of EGFP MYC peaks with published MYC peaks was determined by DiffBind^53^ with default parameters. The heatmaps were generated by the average normalized peak intensity within ±2 kb from peak center with 100 bp bin size. The peak read graph showed the average normalized peak intensity in EGFP, SNF5 and OMOMYC, where peaks were ranked by the normalized intensity in EGFP. GO term analysis was performed on genes assigned to MYC peaks that fell within 1 kb of promoter using functional annotation clustering through DAVID (https://david.ncifcrf.gov/). Correlation of ChIP-Seq replicates on normalized counts of all promoters is presented in Supplementary Fig. 8a.

### ATAC-Seq processing and analysis

Adapter sequences of ATAC-seq reads were trimmed by cutadapt^55^ (cutadapt -a CTGTCTCTTATACACATCT-minimum-length 15 -paired-output), then aligned to the human genome using Bowtie2^51^ (bowtie2 -p 8 -X 2000 -q–no-mixed–no-discordant). Peaks in each sample were called using MACS2 with a *q* value of 0.001 (callpeak -q 0.001 -nomodel -extsize 140)^52^. Peaks were annotated using Homer command annotatePeaks to determine whether peaks were near TSS promoter or far away from TSS (TSS-distal). Enriched motifs were identified by Homer command findMotifsGenome with the default region size and the motif length (-size 200 and -len 8, 10, 12) (http://homer.ucsd.edu/homer/). Consensus peaks in each condition were identified by DiffBind^53^ where peaks occurring at least two replicates were included. Peaks identified across conditions were combined into a final peak set and ATAC-seq read counts for the final peak set were calculated using DiffBind^53^. Read counts were normalized by the RLE method, and differential peaks were identified by DESeq2^54^. The significantly changed peaks were assessed with an FDR < 0.05. GO term analysis was performed on annotated genes (HOMER) that were assigned to gained ATAC-seq peaks following SNF5 reintroduction using functional annotation clustering through DAVID (https://david.ncifcrf.gov/). Correlation of ATAC-Seq replicates on normalized counts of all promoters is presented in Supplementary Fig. 8b.

### PRO-Seq processing and analysis

After adapter trimming and low-quality sequence removal by cutadapt^55^, PRO-seq reads longer than 15 bp were reversed complemented using FastX tools^8^. Reversed complemented reads were aligned to human genome using Bowtie2^51^. Reads mapped to rRNA loci and reads with mapping quality less than 10 were removed. The reads were normalized by the RLE implemented in the DESeq2 ^54^. NRSA (http://bioinfo.vanderbilt.edu/NRSA/), a tool to provide a comprehensive analysis on nascent transcriptional profiles for known genes, was used to estimate RNA polymerase abundance in proximal-promoter and gene body regions of genes, to calculate pausing index and pausing index alterations. Briefly, the promoter-proximal region is defined by examining each 50 bp window with a 5 bp sliding step along the coding strand spanning ± 500 bp from known TSSs. The 50 bp region with the largest number of reads is considered as the promoter-proximal region and its read density is calculated^56^. Gene body is defined as the region from +1 kb downstream of a TSS to its transcription termination site (TTS). Pausing index for each gene is calculated as the ratio of promoter-proximal density over gene body density and the significance of pausing is evaluated by Fisher’s exact test^56^. DESeq2^54^ was implemented to detect significant transcriptional changes for promoter-proximal and gene body regions accounting for the batch effect. The significantly transcriptional changes were assessed with an FDR < 0.05 or <0.0001 as described in figure legends. GO term analysis was performed on the overlapped set of genes with an increased pausing index between SNF5 and OMOMYC using functional annotation clustering through DAVID (https://david.ncifcrf.gov/). Correlation of PRO-Seq replicates on gene body densities is presented in Supplementary Fig. 8c.

### Reporting summary

Further information on research design is available in the Nature Research Reporting Summary linked to this article.

## Supplementary information

Supplementary Information

Reporting Summary

## Data Availability

All sequencing data have been deposited at GEO with the accession number GSE109310. Routine metrics for all next-generation sequencing data are presented in Supplementary Table 2. Any other data supporting the findings in this study are available upon request. Uncropped scans for all blots are presented in Supplementary Fig. 9.
