# Supplementary material for: Inhibition of MYC by the SMARCB1 tumor suppressor
Source: Nat Commun. 2019 May 1;10:2014. doi: 10.1038/s41467-019-10022-5 (PMC6494882; doi:10.1038/s41467-019-10022-5)
Supplement: Supplementary file 1 — Supplementary Information [file 41467_2019_10022_MOESM1_ESM.pdf]

**Supplementary Information**

# Inhibition of MYC by the SMARCB1 tumor suppressor

Weissmiller *et al*.,

# Supplementary Figure 1

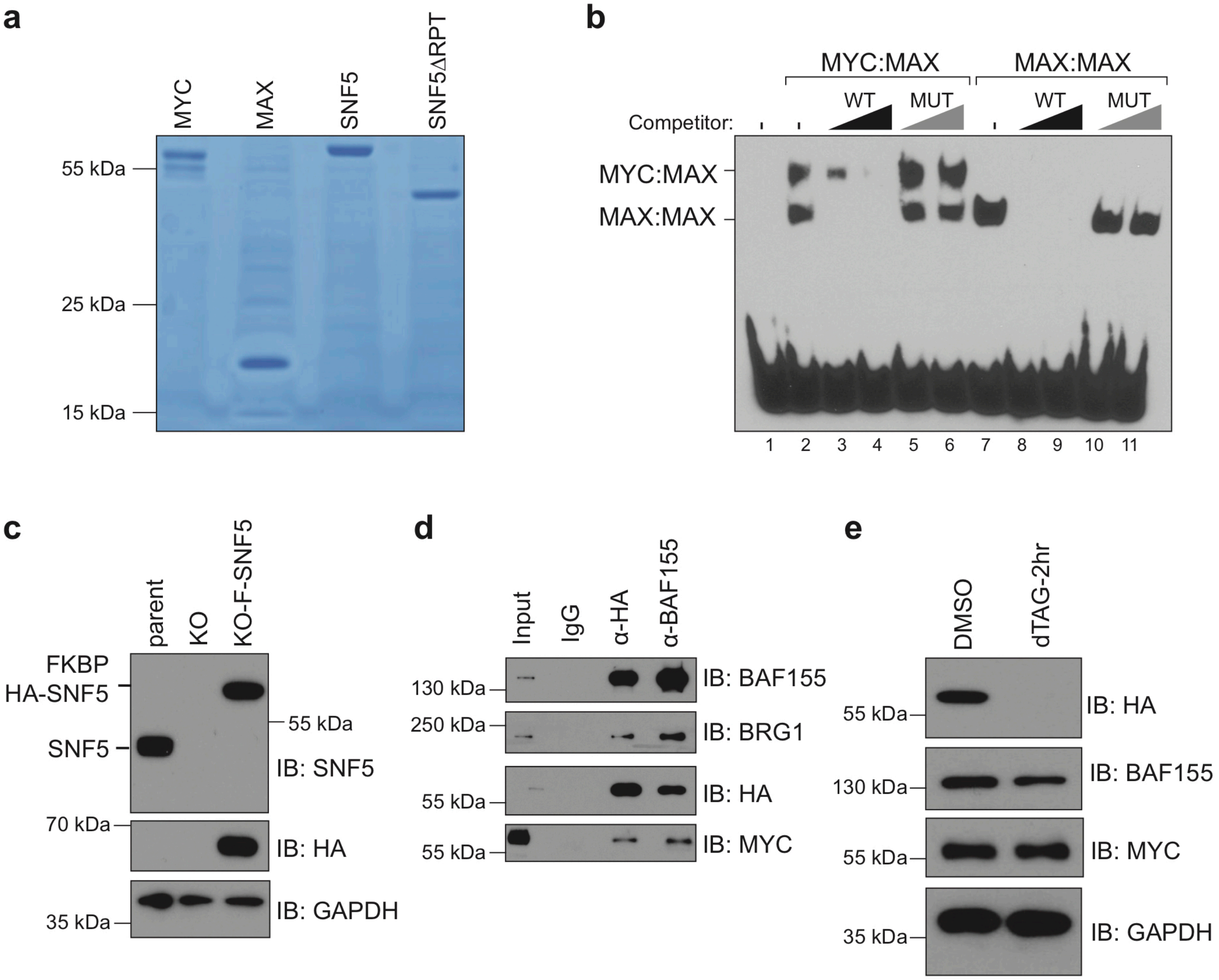


**Supplementary Figure 1.** Validation of experimental systems to look at the impact of SNF5 on DNA-binding by MYC. (a) Commassie stain of recombinant proteins. Sample of MYC and MAX prior to formation of dimers, and recombinant full length SNF or SNF5 in which amino acids containing two conserved imperfect repeats (aa. 176-309) have been removed. (b) Purified recombinant MYC:MAX or MAX:MAX complexes were incubated with a biotinylated double-stranded (ds) DNA probe carrying a wild-type E-box sequence, and DNA binding visualized by an electrophoretic mobility shift assay (EMSA). Triangles indicate 50 or 100 fold molar excess of unlabeled competitor dsDNAs carrying either a wild-type (WT) or mutant 'MUT' E-box sequence. Note that MYC:MAX complexes also contain MAX:MAX homodimers, as indicated. (c) Immunoblot (IB) of lysates from parental HEK293 cells (parent), CRISPR-derived *SMARCB1* null cells (KO), and KO cells reconstituted to express FKBP12(F36V)-HA-tagged SNF5 (KO-F-SNF5). IB was performed with antibodies against SNF5, the HA-tag, or GAPDH (loading control). (d) Lysates were prepared from KO-F-SNF5 cells, immunopreciptated with IgG, an anti-HA, or an anti-BAF155 antibody, and immune complexes probed for the presence of BAF155, BRG1, FKBP12(F36V)-HA-SNF5, or endogenous MYC by immunoblotting. The results show that FKBP12(F36V)-HA-SNF5 interacts with enodgenous SWI/SNF components and MYC. (e) Lysates were prepared from KO-F-SNF5 cells treated with DMSO or 500 nM dTAG-47 for two hours, as indicated.

## Supplementary Figure 2

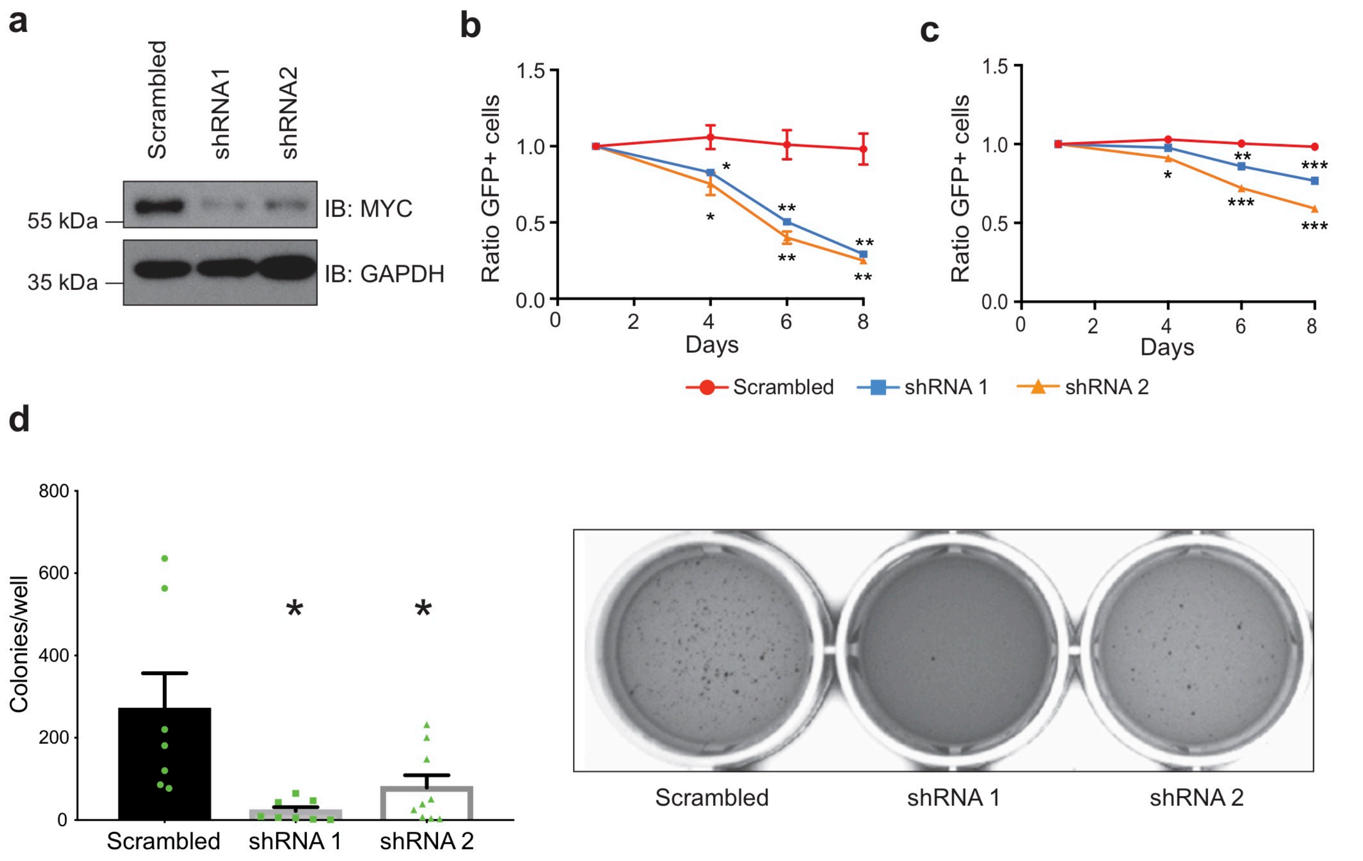


**Supplementary Figure 2.** MYC is important for MRT cell viability. (a) Lysates were collected from HEK293 cells expressing lentiviral shRNAs against MYC or a scrambled shRNA control. Western blot was performed with antibodies against MYC and GAPDH. (b,c) The G401 (b) or A204 (c) MRT cell lines were transduced with the indicated lentiviral shRNA vectors which also co-express the enhanced green fluorescent protein (GFP). The ratio of GFP-positive cells, as judged by flow cytometry, at the indicated times are shown. (n = 3 biological replicates, error bars are standard error; *$p < 0.05$, **$p < 0.01$ ***$p < 0.001$ using unpaired t-test , two-tailed). (d) Anchorage-independent growth in A204 cells transduced with lentiviral shRNAs against MYC or scrambled shRNA control. (n = 3 biological replicates, error bars are standard error; *$p = 0.01$ for shRNA #1 and 0.04 for shRNA #2 using unpaired t-test , two-tailed).

## Supplementary Figure 3

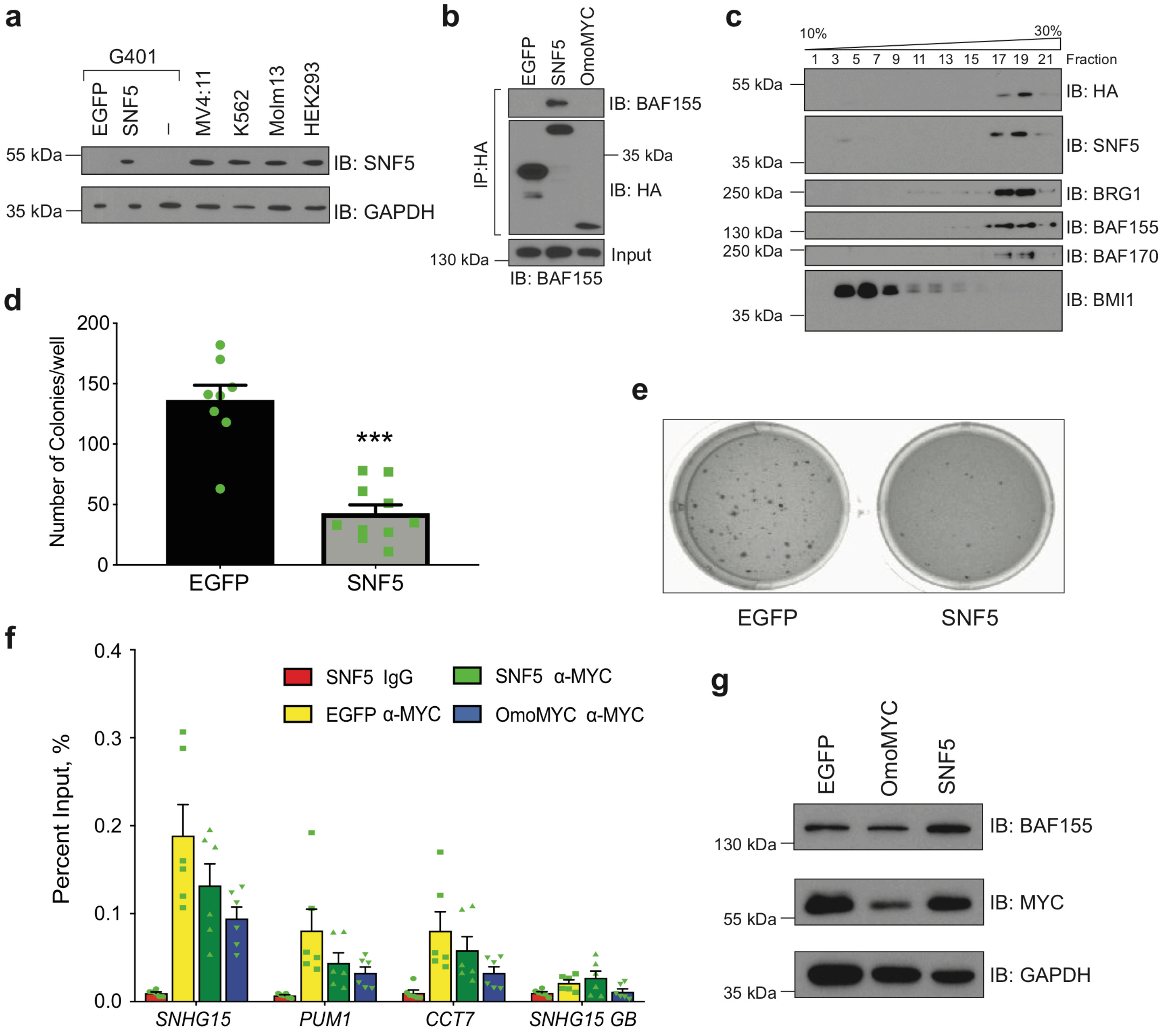


**Supplementary Figure 3.** Validation of experimental MRT cell system. G401 cells were engineered to express doxycycline-inducible versions of HA-epitope tagged EGFP, SNF5, or OmoMYC. After selection, proteins were induced for 24 hours before analysis, unless otherwise indicated. (a) Whole cells lysates from EGFP or SNF5-expressing cells were analyzed against equal amounts of lysate from the indicated cell lines and SNF5 and GAPDH (loading control) levels determined by immunoblotting (IB). (b) Nuclear extracts were collected from G401 cells following expression of HA-SNF5 or HA-OmoMYC. HA-tagged proteins were immunoprecipitated (IP) with an anti-HA antibody under non-denaturing conditions. Immune complexes were then probed for the presence of BAF155 by immunoblot. (c) Glycerol gradient (10-30%) shows assembly of induced SNF5 into large molecular weight complex containing core SWI/SNF subunits. The PRC1 member, BMI1, migrates separately from SWI/SNF and serves as a control. (d) Anchorage-independent growth in G401 cells with chronic induction of SNF5. (n = 3 biological replicates, error bars are standard error; ***$p < 0.001$ using unpaired t-test , two-tailed). (e) Example image from (d). (f) Q-PCR analysis of gene-specific ChIP for MYC binding at three loci in G401 cells engineered to express EGFP, SNF5, or OmoMYC for 24 hours. An antibody against MYC, or IgG control, were used for ChIP. SNHG15 GB is the gene body of SNHG15 and serves as a negative control loci for MYC binding. (n = 6 biological replicates, error bars are standard error). (g) Western blot of lysates prepared following 24hr induction were probed with antibodies against MYC. BAF155 and GAPDH were used as controls.

## Supplementary Figure 4

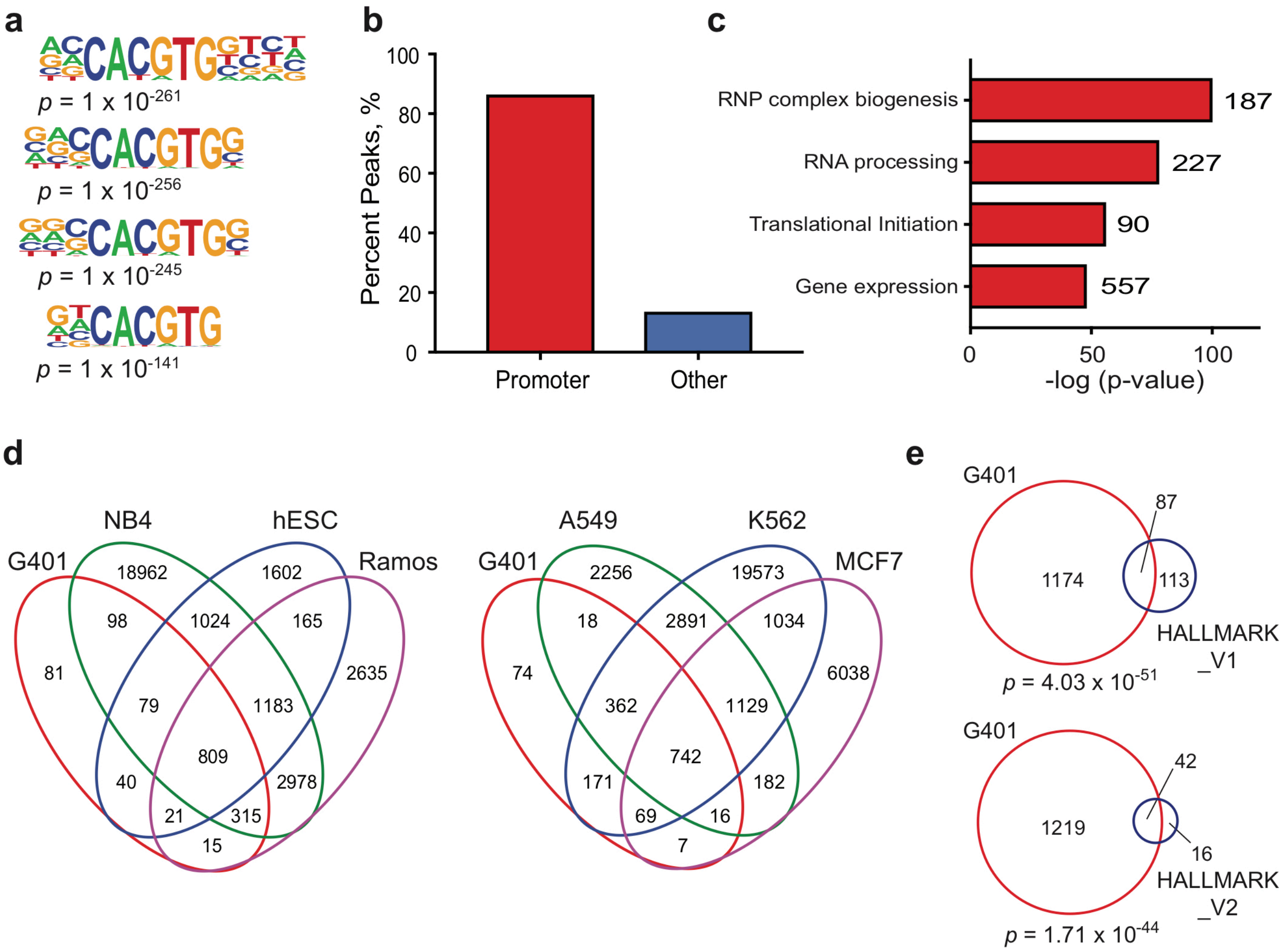


**Supplementary Figure 4.** Relaxed stringency analysis of G401 cell MYC ChIP-Seq. All analyses were performed with an FDR of 0.1. (a) Known motif enrichment analysis was performed on the ChIP-seq data from G401 cells expressing EGFP. The top four motifs are shown; all are enriched in the E-box sequence (CACGTG). (b) MYC peaks in the EGFP-expressing cells were quantified in terms of their distance to the nearest annotated transcriptional start-site (TSS). Those peaks within 1 kb of a TSS were called "promoter". (c) MYC peaks located within 1 kb of a TSS were assigned to their nearest gene and GO term enrichment analysis was performed. (d) Overlap of MYC peaks obtained from EGFP-expressing G401 cells with six published MYC ChIP-seq data sets: NB4 acute promyelocytic leukemia cells (GSM935643), hESC human embryonic stem cells (GSM935509), Ramos Burkitt's lymphoma cells (GSM762711), A549 lung cancer cells (GSM1003607), K562 chronic myeloid leukemia cells (GSM935516), and MCF7 breast cancer cells (GSM1006866). (e) MYC peaks that are located within 1kb from a TSS were assigned to their nearest gene and overlaid with two MSigDB Hallmark MYC target data sets. A hypergeometric test was performed; significance is displayed below the Venn diagram. n = 2 independent ChIP-seq experiments.

## Supplementary Figure 5

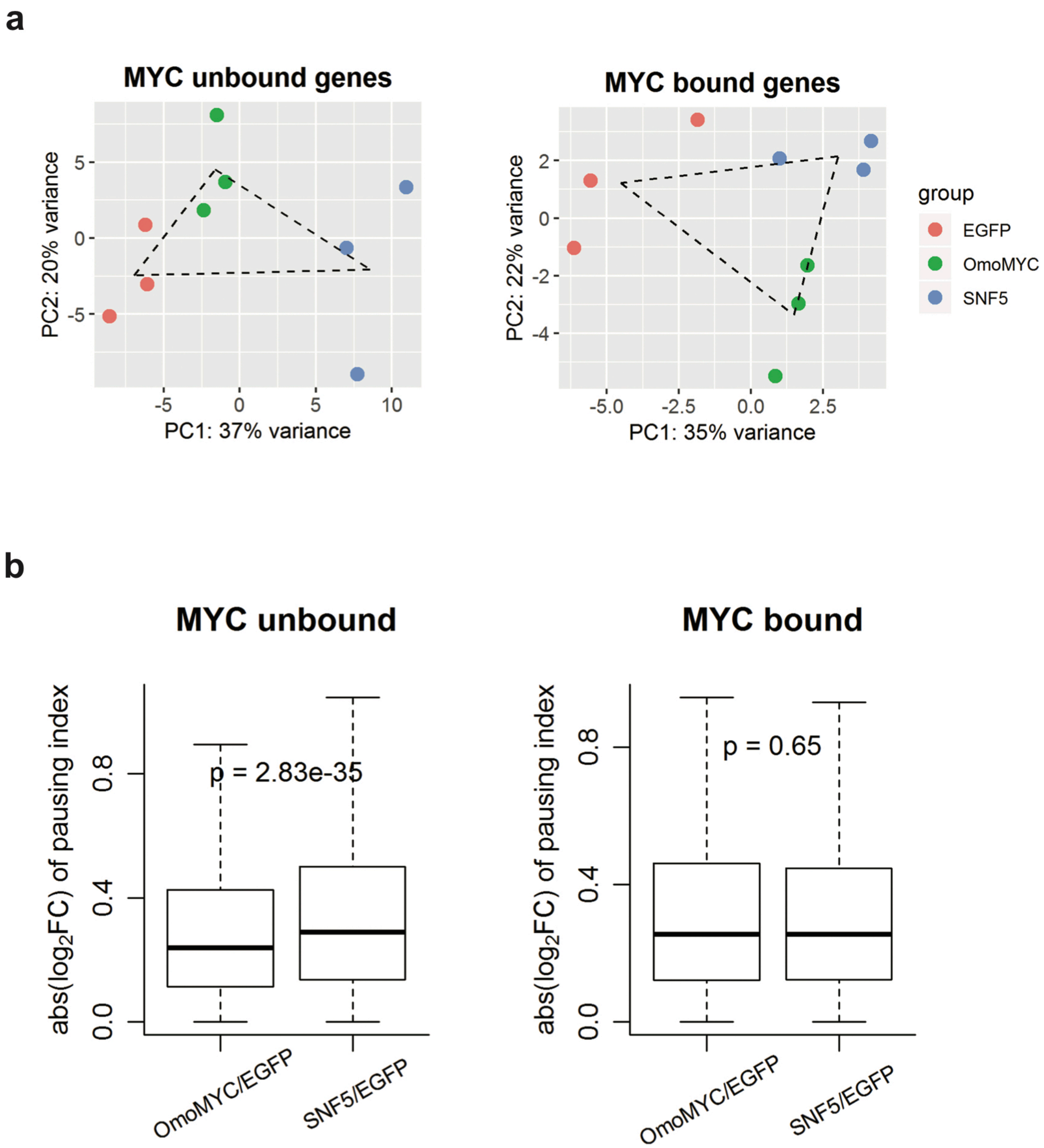


**Supplementary Figure 5.** Effect of SNF5 on RNA polymerase pausing mimics that of MYC inhibition. (a) Principle component analysis of pausing indices of EGFP, OmoMYC, and SNF5 on MYC unbound genes (left) and bound ones (right). Distances between the centroids of each group are indicated in dashed lines. OmoMYC is closer to EGFP than to SNF5 in MYC unbound genes (t-test, p-value = 0.0006), while OmoMYC is closer to SNF5 in MYC bound genes (t-test, p-value = 0.016). (b) Boxplots of pausing indices differences of OmoMYC and SNF5, compared to EGFP, measured by absolute value of log2-fold change (abs(log2FC)), in MYC unbound (left) and MYC bound (right) genes. Box extends from the first quartile (25th percentile) to the third quartile (75th percentile) with median marked by the middle line; whiskers extend from minimum to maximum point.

## Supplementary Figure 6

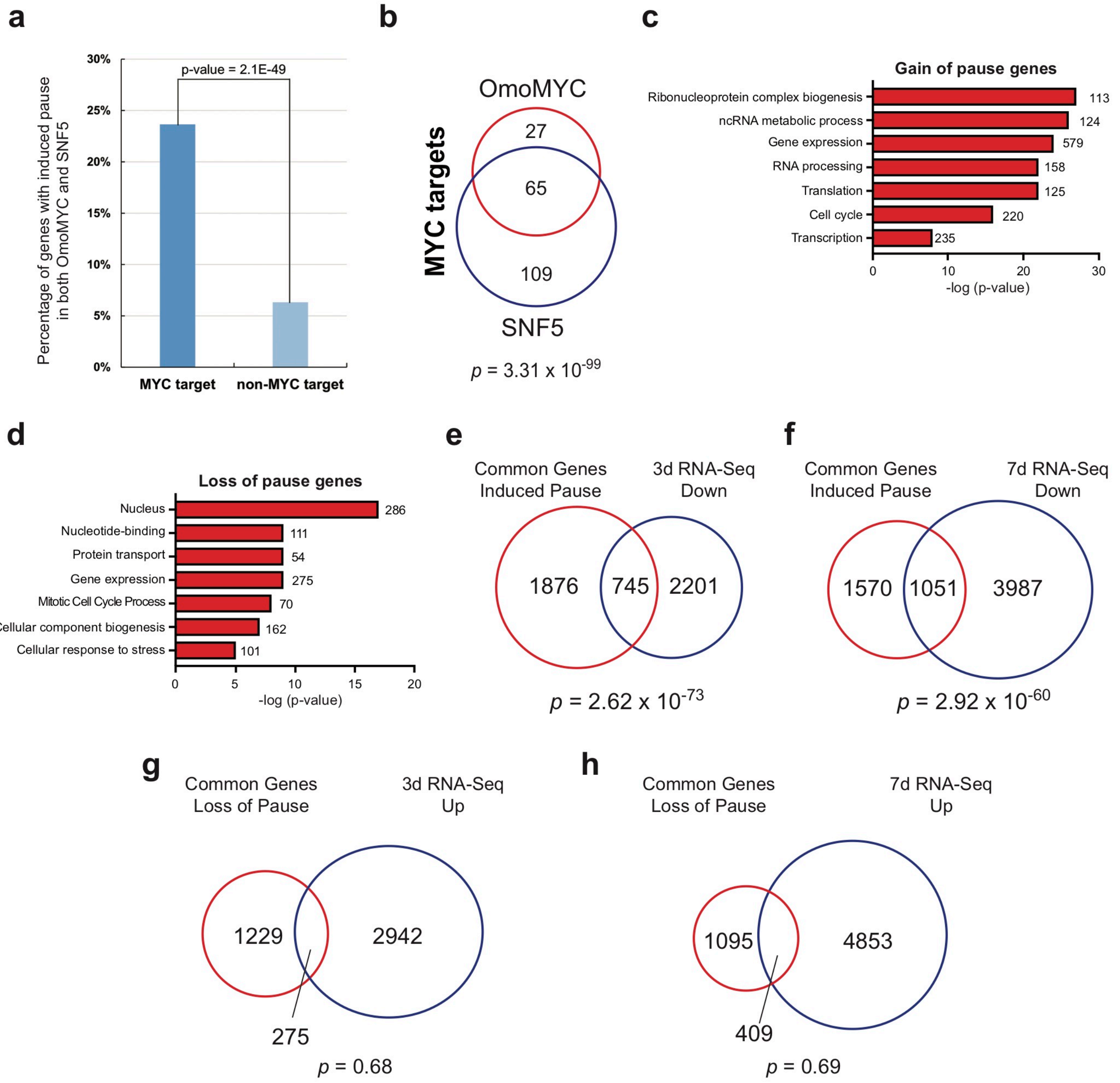


**Supplementary Figure 6.** Analysis of gene expression changes in G401 cells, as measured by PRO-Seq. (a) Proportion of MYC-target or non-target genes with induced pause in both OmoMYC and SNF5. MYC targets have a statistically higher overlap with the genes in which pause was induced in both OmoMYC and SNF5 than non-targets (Fisher's exact test, p-value = 2.1E-49). (b) Overlap between the number of genes identified as MYC targets from ChIP-seq analysis that also had a resulting loss of pause for each condition. (c) GO term analysis of genes with a gain of RNA polymerase pause (FDR < 0.0001) that were shared between OmoMYC and SNF5. (d) GO term analysis of genes with a loss of RNA polymerase pause (FDR < 0.0001) that were shared between OmoMYC and SNF5. (e) Overlap of genes showing a common OmoMYC/SNF5-induced pause (FDR < 0.05) with RNA-Seq data (GSE90633) showing decreased gene expression changes in G401 cells after three days of SNF5 expression. The overlap is highly significant. (f) Overlap of genes showing a common OmoMYC/SNF5-induced pause (FDR < 0.05) with RNA-Seq data (GSE90633) showing decreased gene expression changes in G401 cells after seven days of SNF5 expression. The overlap is highly significant. (g) Overlap of genes showing a common OmoMYC/SNF5-lost pause (FDR < 0.05) with RNA-Seq data (GSE90633) showing increased gene expression changes in G401 cells after three days of SNF5 expression. The overlap is not significant. (h) Overlap of genes showing a common OmoMYC/SNF5-lost pause with RNA-Seq data (GSE90633) showing increased gene expression changes in G401 cells after seven days of SNF5 expression. The overlap is not significant. n = 3 independent PRO-Seq experiments.

# Supplementary Figure 7

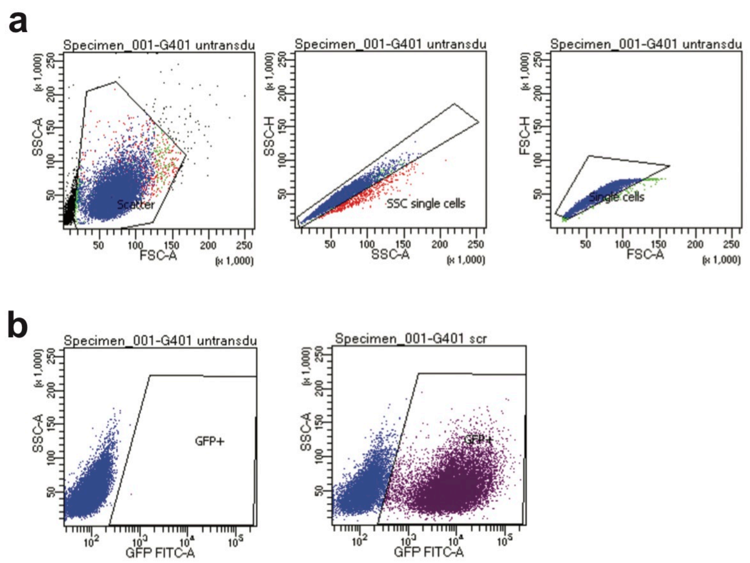


**Supplementary Figure 7.** Gating strategy used for flow cytometry. Representative data are shown. (a) Single G401 (shown) or A204 cells were selected using forward and side scatter as outlined. (b) To count GFP-expressing cells, an untransduced cell line control (left) was measured to determine baseline fluorescence, as outlined. GFP-positive cells were scored in the indicated sector, based on gating against the untransduced cell line control. An example GFP-positive cell line is shown (right).

## Supplementary Figure 8

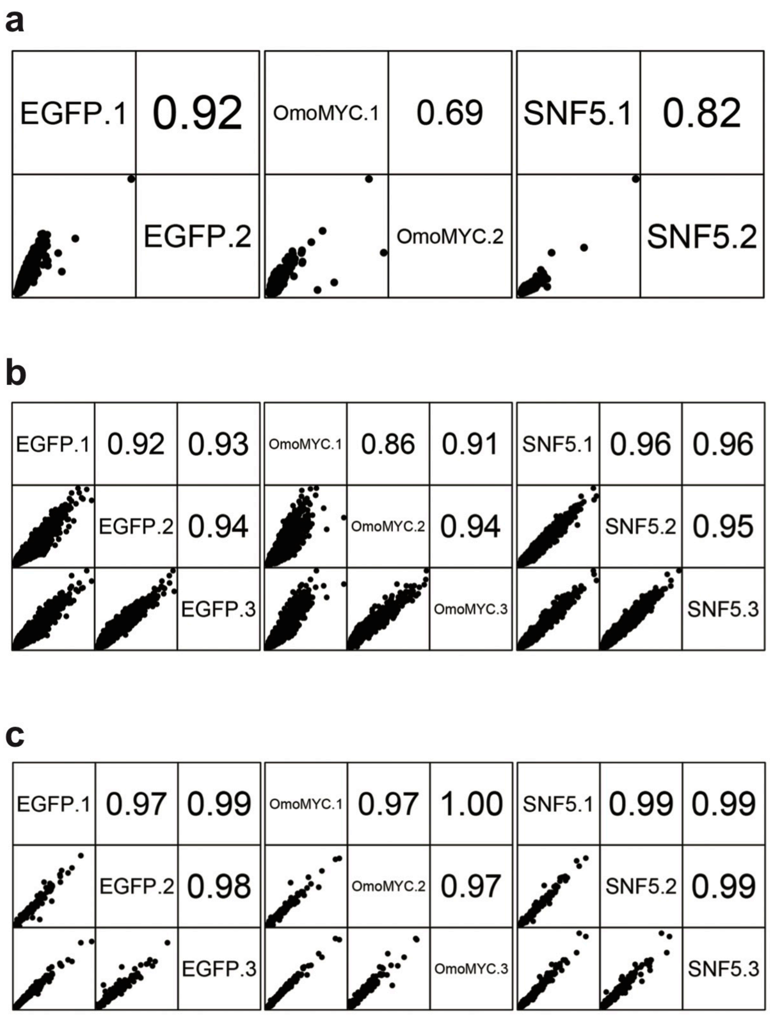


**Supplementary Figure 8.** Biological replicates are well correlated in ChIP-Seq, ATAC-Seq, and PRO-Seq data. Correlation on normalized counts of all promoters for ChIP-Seq (a) and ATAC-Seq replicates (b), and on gene-body densities for PRO-Seq replicates (c). Lower panels are the pairwise scatter plots of the replicates on normalized counts, and upper panels are the pairwise correlation coefficient of the replicates. The diagonal displays the replicate labels.

# Supplementary Figure 9

**Fig 1a**

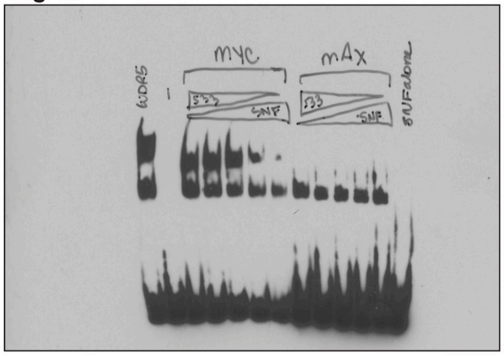


**Fig 1b**

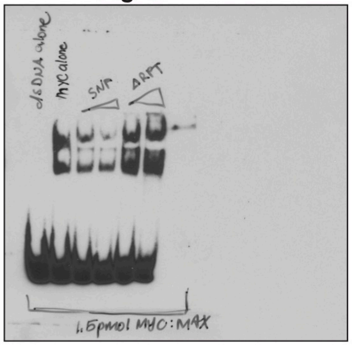


**Sup Fig 1a**

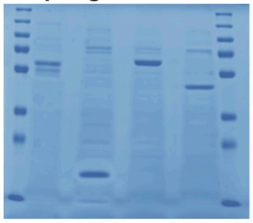

**Fig 1c**

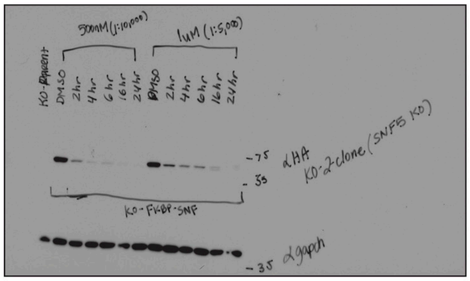


**Sup Fig 1b**

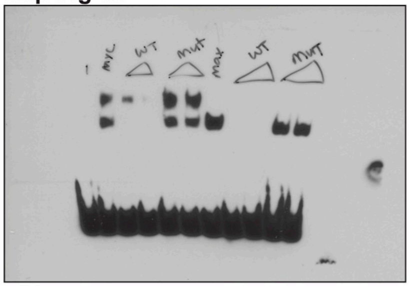


**Sup Fig 1c**

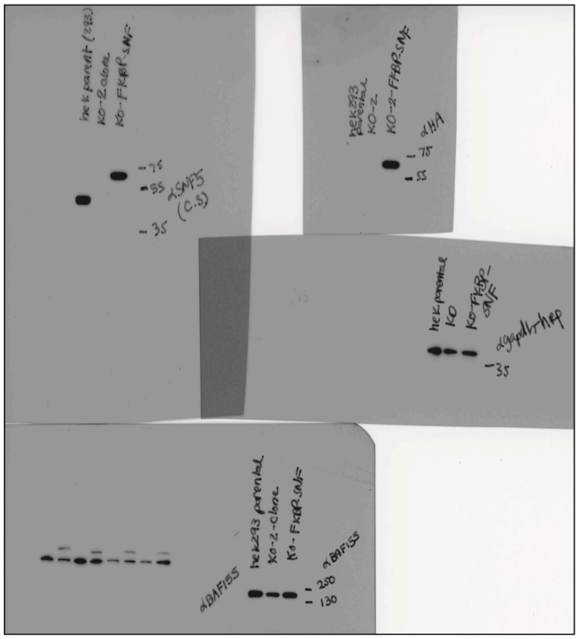


**Sup Fig 1d**

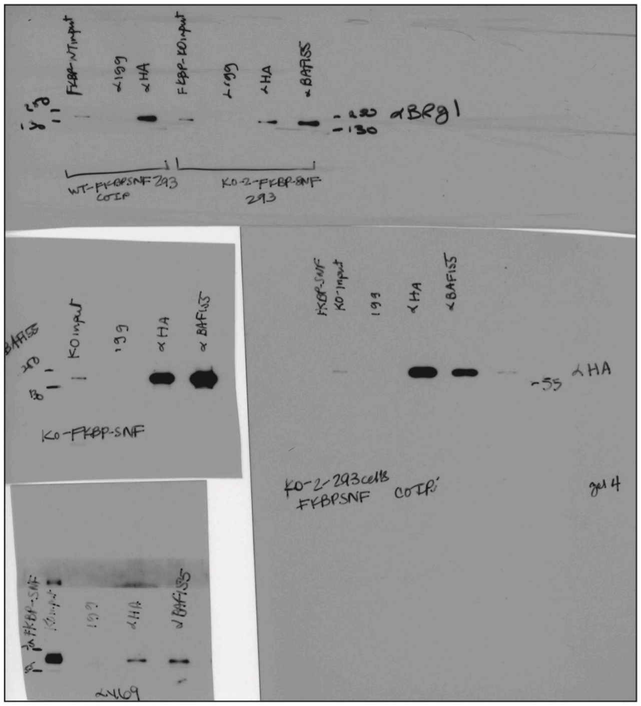


**Supplementary Figure 9–Part 1.** Untrimmed immunoblot gels. Each untrimmed immunoblot is identified by the their figure label within the text and are outlined together. If there are multiple immunoblots per figure section, they have been labelled similarly.

# Supplementary Figure 9

Sup Fig 1e

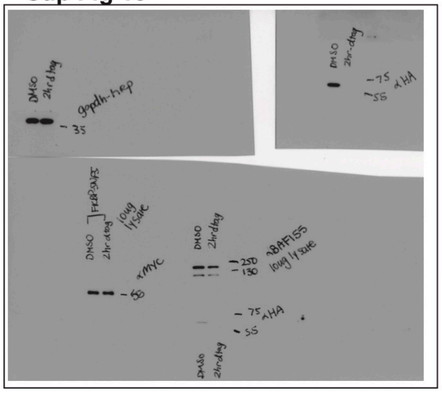

Sup Fig 2a

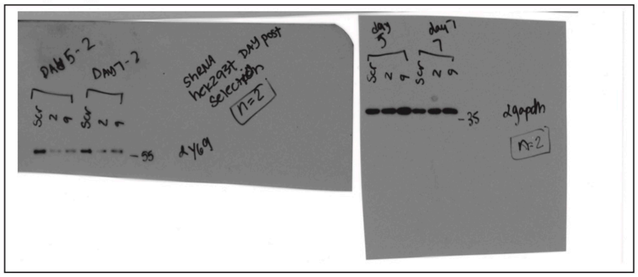

Sup Fig 3a

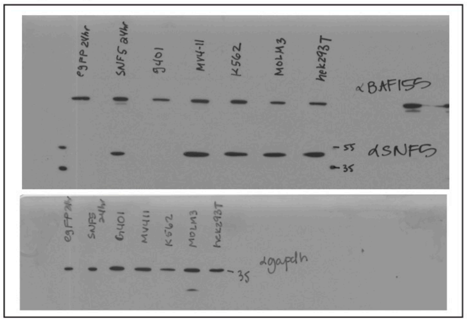

Sup Fig 3b

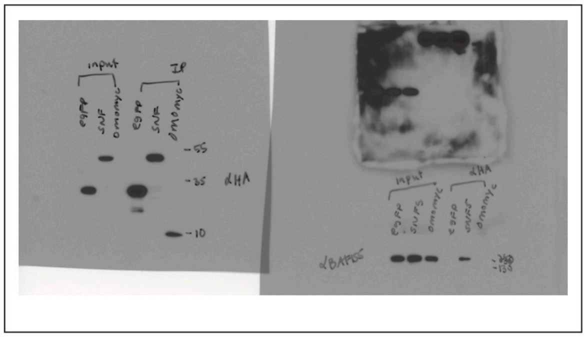

Sup Fig 3g

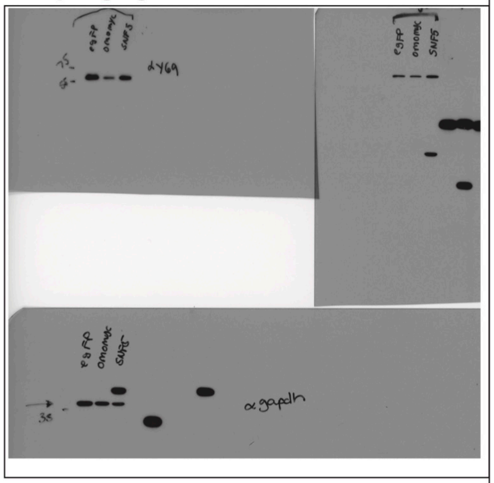

Sup Fig 3c

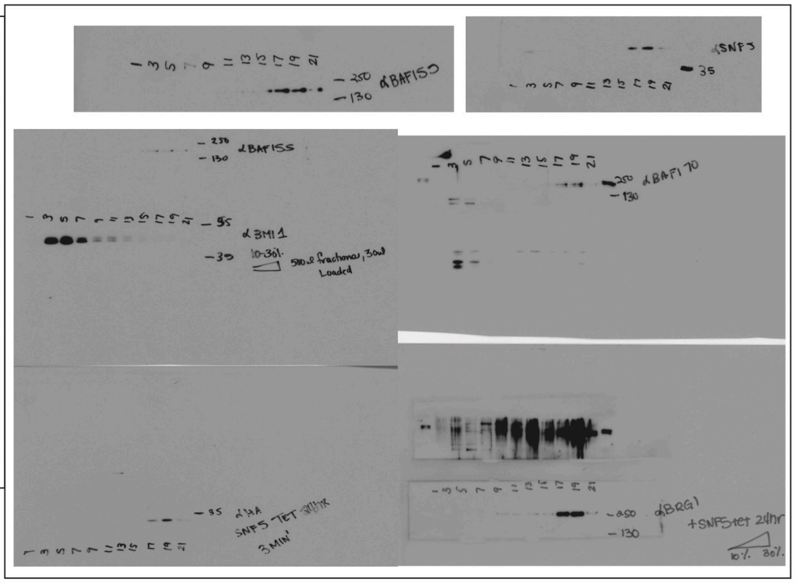

**Supplementary Figure 9–Part 2.** Untrimmed immunoblot gels. Each untrimmed immunoblot is identified by the their figure label within the text and are outlined together. If there are multiple immunoblots per figure section, they have been labelled similarly.

**Supplementary Table 1. Top 20 known motifs for ATAC-Seq peaks gained upon SNF5 reintroduction.**

| HOMER-Known Motif Results (Top 20) | | | |
|---|---|---|---|
| **Motif Name** | **Consensus** | **P-value** | **# of Target Sequences with Motif** |
| BATF(bZIP)/Th17-BATF-ChIP-Seq(GSE39756)/Homer | DATGASTCAT | 1e-1016 | 1494 |
| Atf3(bZIP)/GBM-ATF3-ChIP-Seq(GSE33912)/Homer | DATGASTCATHN | 1e-1015 | 1505 |
| Jun-AP1(bZIP)/K562-cJun-ChIP-Seq(GSE31477)/Homer | GATGASTCATCN | 1e-971 | 1046 |
| AP-1(bZIP)/ThioMac-PU.1-ChIP-Seq(GSE21512)/Homer | VTGACTCATC | 1e-953 | 1511 |
| Bach2(bZIP)/OCILy7-Bach2-ChIP-Seq(GSE44420)/Homer | TGCTGAGTCA | 1e-513 | 652 |
| TEAD4(TEA)/Tropoblast-Tead4-ChIP-Seq(GSE37350)/Homer | CCWGGAATGY | 1.00E-210 | 787 |
| NF-E2(bZIP)/K562-NFE2-ChIP-Seq(GSE31477)/Homer | GATGACTCAGCA | 1.00E-201 | 231 |
| TEAD(TEA)/Fibroblast-PU.1-ChIP-Seq(Unpublished)/Homer | YCWGGAATGY | 1.00E-200 | 693 |
| Bach1(bZIP)/K562-Bach1-ChIP-Seq(GSE31477)/Homer | AWWNTGCTGAGTCAT | 1.00E-190 | 217 |
| Nrf2(bZIP)/Lymphoblast-Nrf2-ChIP-Seq(GSE37589)/Homer | HTGCTGAGTCAT | 1.00E-167 | 187 |
| MafK(bZIP)/C2C12-MafK-ChIP-Seq(GSE36030)/Homer | GCTGASTCAGCA | 1.00E-160 | 357 |
| MafA(bZIP)/Islet-MafA-ChIP-Seq(GSE30298)/Homer | TGCTGACTCA | 1.00E-95 | 528 |
| Lhx2(Homeobox)/HFSC-Lhx2-ChIP-Seq(GSE48068)/Homer | TAATTAGN | 1.00E-53 | 537 |
| Lhx3(Homeobox)/Neuron-Lhx3-ChIP-Seq(GSE31456)/Homer | ADBTAATTAR | 1.00E-49 | 742 |
| Nkx6.1(Homeobox)/Islet-Nkx6.1-ChIP-Seq(GSE40975)/Homer | GKTAATGR | 1.00E-42 | 1024 |
| Pdx1(Homeobox)/Islet-Pdx1-ChIP-Seq(SRA008281)/Homer | YCATYAATCA | 1.00E-38 | 450 |
| Unknown-ESC-element/mES-Nanog-ChIP-Seq(GSE11724)/Homer | CACAGCAGGGGG | 1.00E-34 | 313 |
| Isl1(Homeobox)/Neuron-Isl1-ChIP-Seq(GSE31456)/Homer | CTAATKGV | 1.00E-33 | 783 |
| Nanog(Homeobox)/mES-Nanog-ChIP-Seq(GSE11724)/Homer | RGCCATTAAC | 1.00E-33 | 1448 |
| FOXA1(Forkhead)/MCF7-FOXA1-ChIP-Seq(GSE26831)/Homer | WAAGTAAACA | 1.00E-32 | 408 |

**Supplementary Table 2. NGS metrics**

| Sample | Total reads | Total mapped reads | Uniquely mapped reads | Percent of uniquely mapped reads | Mapped reads after removing PCR duplicates |
|---|---|---|---|---|---|
| ChIP-seq IgG MYC EGFP rep1 | 71717356 | 69335593 | 51969683 | 72.46% | 43969047 |
| ChIP-seq IgG MYC EGFP rep2 | 73602197 | 71599163 | 53421723 | 72.58% | 50176484 |
| ChIP-seq IgG MYC OMOMYC rep1 | 71717356 | 69335593 | 51969683 | 72.46% | 43969047 |
| ChIP-seq IgG MYC OMOMYC rep2 | 89589956 | 86816147 | 65245771 | 72.83% | 41234974 |
| ChIP-seq IgG MYC SNF5 rep1 | 71717356 | 69335593 | 51969683 | 72.46% | 43969047 |
| ChIP-seq IgG MYC SNF5 rep2 | 73602197 | 71599163 | 53421723 | 72.58% | 50176484 |
| ChIP-seq MYC EGFP rep1 | 77398344 | 75204491 | 56729420 | 73.30% | 61613363 |
| ChIP-seq MYC EGFP rep2 | 96154783 | 94046624 | 72720124 | 75.63% | 72222784 |
| ChIP-seq MYC OMOMYC rep1 | 71834322 | 69850089 | 53901402 | 75.04% | 48038862 |
| ChIP-seq MYC OMOMYC rep2 | 70344428 | 68566129 | 52871427 | 75.16% | 55611783 |
| ChIP-seq MYC SNF5 rep1 | 67851301 | 65957113 | 49709443 | 73.26% | 44167648 |
| ChIP-seq MYC SNF5 rep2 | 78693413 | 76205871 | 57049492 | 72.50% | 56012175 |
| ATAC-seq EGFP rep1 | 90635977 | 76516845 | 53525433 | 59.06% | 37406714 |
| ATAC-seq EGFP rep2 | 124361328 | 87340595 | 61730054 | 49.64% | 44295744 |
| ATAC-seq EGFP rep3 | 136557636 | 121597994 | 81444637 | 59.64% | 75184769 |
| ATAC-seq OMOMYC rep1 | 97150468 | 77213754 | 53346742 | 54.91% | 39530431 |
| ATAC-seq OMOMYC rep2 | 130620417 | 87625295 | 60221032 | 46.10% | 51760437 |
| ATAC-seq OMOMYC rep3 | 138942753 | 119290812 | 80669479 | 58.06% | 70428103 |
| ATAC-seq SNF5 rep1 | 105503925 | 89754487 | 63009713 | 59.72% | 45935898 |
| ATAC-seq SNF5 rep2 | 140022078 | 106282258 | 74208597 | 53.00% | 64906362 |
| ATAC-seq SNF5 rep3 | 124033352 | 113376174 | 77180119 | 62.23% | 77448512 |
| PRO-seq EGFP rep1 | 90635977 | 76516845 | 53525433 | 59.06% | 33140211 |
| PRO-seq EGFP rep2 | 124361328 | 87340595 | 61730054 | 49.64% | 52133601 |
| PRO-seq EGFP rep3 | 136557636 | 121597994 | 81444637 | 59.64% | 70048835 |
| PRO-seq OMOMYC rep1 | 97150468 | 77213754 | 53346742 | 54.91% | 33261214 |
| PRO-seq OMOMYC rep2 | 130620417 | 87625295 | 60221032 | 46.10% | 48376961 |
| PRO-seq OMOMYC rep3 | 138942753 | 119290812 | 80669479 | 58.06% | 60863604 |
| PRO-seq SNF5 rep1 | 105503925 | 89754487 | 63009713 | 59.72% | 41923693 |
| PRO-seq SNF5 rep2 | 140022078 | 106282258 | 74208597 | 53.00% | 63178107 |
| PRO-seq SNF5 rep3 | 124033352 | 113376174 | 77180119 | 62.23% | 83908119 |
