# Supplementary material for: Inhibition of MYC by the SMARCB1 tumor suppressor
Source: Nat Commun. 2019 May 1;10:2014. doi: 10.1038/s41467-019-10022-5 (PMC6494882; doi:10.1038/s41467-019-10022-5)
Supplement: Supplementary file 2 — Reporting Summary [file 41467_2019_10022_MOESM2_ESM.pdf]

nature research

Corresponding author(s): William P. Tansey

Last updated by author(s): Mar 28, 2019

Reporting Summary

Nature Research wishes to improve the reproducibility of the work that we publish. This form provides structure for consistency and transparency in reporting. For further information on Nature Research policies, see Authors & Referees and the Editorial Policy Checklist.

Statistics

For all statistical analyses, confirm that the following items are present in the figure legend, table legend, main text, or Methods section.

n/a	Confirmed	
☐	☒	The exact sample size (*n*) for each experimental group/condition, given as a discrete number and unit of measurement
☐	☒	A statement on whether measurements were taken from distinct samples or whether the same sample was measured repeatedly
☐	☒	The statistical test(s) used AND whether they are one- or two-sided<br>*Only common tests should be described solely by name; describe more complex techniques in the Methods section.*
☒	☐	A description of all covariates tested
☐	☒	A description of any assumptions or corrections, such as tests of normality and adjustment for multiple comparisons
☐	☒	A full description of the statistical parameters including central tendency (e.g. means) or other basic estimates (e.g. regression coefficient) AND variation (e.g. standard deviation) or associated estimates of uncertainty (e.g. confidence intervals)
☐	☒	For null hypothesis testing, the test statistic (e.g. *F*, *t*, *r*) with confidence intervals, effect sizes, degrees of freedom and *P* value noted<br>*Give P values as exact values whenever suitable.*
☒	☐	For Bayesian analysis, information on the choice of priors and Markov chain Monte Carlo settings
☒	☐	For hierarchical and complex designs, identification of the appropriate level for tests and full reporting of outcomes
☐	☒	Estimates of effect sizes (e.g. Cohen's *d*, Pearson's *r*), indicating how they were calculated

Our web collection on statistics for biologists contains articles on many of the points above.

Software and code

Policy information about availability of computer code

Data collection	ChIP-Seq: Bowtie 2 - version 2.3.4.1, MACS2 - version 2.1.1.20160309, DiffBind - version 1.12.3.<br>PRO-Seq: FASTX-Toolkit - version 0.0.14, Bowtie 2 - version 2.3.4.1, NRSA - http://bioinfo.vanderbilt.edu/NRSA/<br>ATAC-Seq: Cutadapt - version 1.18, Bowtie 2 - version 2.3.4.1, MACS2 - version 2.1.1.20160309, HOMER - version 4.10, DiffBind - version 1.12.3.
Data analysis	After adapter trimming and low quality sequence removal by cutadapt, PRO-Seq reads longer than 15bp were reversed complemented using FastX tools. Reverse complements of the trimmed reads were aligned to the human genome hg19 using Bowtie2. Reads mapped to rRNA loci and reads with mapping quality less than 10 were removed. Bam files were given to NRSA (http://bioinfo.vanderbilt.edu/NRSA/) to estimate RNA polymerase abundance in proximal-promoter and gene body regions of genes, to calculate pausing index and pausing index alterations, and to detect enhancers and quantify eRNA changes.<br>ChIP-Seq reads were aligned to the hg19 genome assembly using Bowtie2. Peaks were called by MACS2 with a q-value of 0.01. Differential binding peaks were identified using DiffBind based on consensus peaks occurring at least two samples.<br>Adapter sequences of ATAC-Seq reads were trimmed by Cutadapt (cutadapt -a CTGTCTCTTA TACACATCT-minimum-length 15 -paired-output), then aligned to the human genome hg38 using Bowtie2 (bowtie2 -p 8 -X 2000 -q --no-mixed --no-discordant). Peaks were called using MACS2 with q-value of 0.001 (callpeak -q 0.001 -nomodel -extsize 140). Peaks were annotated and enriched motifs were identified by HOMER. Differential enriched peaks were identified using DiffBind based on consensus peaks occurring at least two samples.

For manuscripts utilizing custom algorithms or software that are central to the research but not yet described in published literature, software must be made available to editors/reviewers. We strongly encourage code deposition in a community repository (e.g. GitHub). See the Nature Research guidelines for submitting code & software for further information.

Data

Policy information about availability of data

All manuscripts must include a data availability statement. This statement should provide the following information, where applicable:

- Accession codes, unique identifiers, or web links for publicly available datasets
- A list of figures that have associated raw data
- A description of any restrictions on data availability

Data Availability. All sequencing data have been deposited at GEO with the accession number GSE109310 [https://www.ncbi.nlm.nih.gov/geo/query/acc.cgi?acc=GSE109310]. Routine metrics for all next generation sequencing (NGS) data are presented in Supplementary Table 2. Any other data supporting the findings in this study are available upon request. Uncropped scans for all blots are presented in Supplementary Figure 9.

Field-specific reporting

Please select the one below that is the best fit for your research. If you are not sure, read the appropriate sections before making your selection.

☒ Life sciences ☐ Behavioural & social sciences ☐ Ecological, evolutionary & environmental sciences

For a reference copy of the document with all sections, see nature.com/documents/nr-reporting-summary-flat.pdf

Life sciences study design

All studies must disclose on these points even when the disclosure is negative.

Sample size	No statistical methods were used to determine the sample sizes. Our sample sizes are similar to those generally employed in the field and as extensively used in our previously published studies. Typically three biological replicates were used in our studies. The exact n values for each experiment can be found in the figure legends.
Data exclusions	No data were excluded from this analysis.
Replication	All replication attempts were successful. The number of times an experiment was repeated (biological replicates) and exact n values are indicated in the figure legends and/or methods section.
Randomization	Randomization of samples in groups is not relevant to this study as no animal or clinical work was performed.
Blinding	For soft agar assays, the investigator counting colonies was blinded to the identification of each well.

Reporting for specific materials, systems and methods

We require information from authors about some types of materials, experimental systems and methods used in many studies. Here, indicate whether each material, system or method listed is relevant to your study. If you are not sure if a list item applies to your research, read the appropriate section before selecting a response.

Materials & experimental systems

n/a	Involved in the study	
☐	☒	Antibodies
☐	☒	Eukaryotic cell lines
☒	☐	Palaeontology
☒	☐	Animals and other organisms
☒	☐	Human research participants
☒	☐	Clinical data

Methods

n/a	Involved in the study	
☐	☒	ChIP-seq
☐	☒	Flow cytometry
☒	☐	MRI-based neuroimaging

Antibodies

Antibodies used	For Western blotting: SNF5 (Bethyl Laboratories, A301-087A, Abcam, ab12167, and Cell Signaling, 91735), BAF155 (Cell Signaling, D7F8; 1:1000), GAPDH-HRP (Invitrogen, MA5-15738), HA-epitope tag (Cell Signaling, C29F4), HA-HRP (Roche, 12013819001), MYC (Santa Cruz Biotechnology, sc-274), and MAX (Santa Cruz Biotechnology, sc-275). For ChIP, MYC (N262, Santa Cruz Biotechnology, sc-764 or Cell Signaling, 9402) or normal rabbit IgG control (Cell Signaling, 2729S)
Validation	Details for validation are available on the manufacturers' websites.

Eukaryotic cell lines

Policy information about cell lines

Cell line source(s)	All cell lines were obtained from the ATCC
Authentication	ATCC Cell Line Authentication Kit
Mycoplasma contamination	Cell lines were tested for mycoplasma contamination using the PCR Mycoplasma Detection Kit (abm, Cat.#G238), and were negative.
Commonly misidentified lines (See ICLAC register)	None

ChIP-seq

Data deposition

☒ Confirm that both raw and final processed data have been deposited in a public database such as GEO.

☐ Confirm that you have deposited or provided access to graph files (e.g. BED files) for the called peaks.

Data access links *May remain private before publication.*	Both raw data and the final processed peak files for ChIP-Seq have been deposited at the Gene Expression Omnibus (GEO) with accession number GSE109310.
Files in database submission	ChIPseq-EGFP-1_peaks.narrowPeak.gz<br>ChIPseq-EGFP-2_peaks.narrowPeak.gz<br>ChIPseq-OMOMYC-1_peaks.narrowPeak.gz<br>ChIPseq-OMOMYC-2_peaks.narrowPeak.gz<br>ChIPseq-SNF5-1_peaks.narrowPeak.gz<br>ChIPseq-SNF5-2_peaks.narrowPeak.gz<br>ATACseq-EGFP-1_peaks.narrowPeak.gz<br>ATACseq-EGFP-2_peaks.narrowPeak.gz<br>ATACseq-EGFP-3_peaks.narrowPeak.gz<br>ATACseq-OMOMYC-1_peaks.narrowPeak.gz<br>ATACseq-OMOMYC-2_peaks.narrowPeak.gz<br>ATACseq-OMOMYC-3_peaks.narrowPeak.gz<br>ATACseq-SNF5-1_peaks.narrowPeak.gz<br>ATACseq-SNF5-2_peaks.narrowPeak.gz<br>ATACseq-SNF5-3_peaks.narrowPeak.gz
Genome browser session (e.g. UCSC)	No longer applicable.

Methodology

Replicates	ChIP-Seq were performed with two biological replicates. Peaks of each replicate are enriched in promoter and 5' UTR regions. Peaks were extensively validate using ChIPqPCR. The replicates are well correlated.
Sequencing depth	IgG MYC EGFP rep1: total number of reads - 71717356, uniquely mapped reads - 51969683, length of reads - 75, single-end.<br>IgG MYC EGFP rep2: total number of reads - 73602197, uniquely mapped reads - 53421723, length of reads - 75, single-end.<br>IgG MYC OMOMYC rep1: total number of reads - 71717356, uniquely mapped reads - 51969683, length of reads - 75, single-end.<br>IgG MYC OMOMYC rep2: total number of reads - 89589956, uniquely mapped reads - 65245771, length of reads - 75, single-end.<br>IgG MYC SNF5 rep1: total number of reads - 71717356, uniquely mapped reads - 51969683, length of reads - 75, single-end.<br>IgG MYC SNF5 rep2: total number of reads - 73602197, uniquely mapped reads - 53421723, length of reads - 75, single-end.<br>MYC EGFP rep1: total number of reads - 77398344, uniquely mapped reads - 56729420, length of reads - 75, single-end.<br>MYC EGFP rep2: total number of reads - 96154783, uniquely mapped reads - 72720124, length of reads - 75, single-end.<br>MYC OMOMYC rep1: total number of reads - 71834322, uniquely mapped reads - 53901402, length of reads - 75, single-end.<br>MYC OMOMYC rep2: total number of reads - 70344428, uniquely mapped reads - 52871427, length of reads - 75, single-end.<br>MYC SNF5 rep1: total number of reads - 67851301, uniquely mapped reads - 49709443, length of reads - 75, single-end.<br>MYC SNF5 rep2: total number of reads - 78693413, uniquely mapped reads - 57049492, length of reads - 75, single-end.
Antibodies	For ChIP, MYC (N262, Santa Cruz Biotechnology, sc-764 or Cell Signaling, 9402) or normal rabbit IgG control (Cell Signaling, 2729S)
Peak calling parameters	Peaks in each sample were called using MACS2 with q-value of 0.01 (Feng et al., 2012). Peaks were annotated using the HOMER command annotatePeaks (http://homer.ucsd.edu/homer/). Consensus peaks in each condition were identified using DiffBind [Stark, R. & Brown, G.D. DiffBind: differential binding analysis of ChIP-seq peak data. (Bioconductor, 2011)]; peaks occurring at both replicates in each condition were included. Peaks identified in at least one condition were combined into a final peak set to identify differential peaks across conditions. Read counts were normalized to the total mapped reads,

	and differential peaks were determined by DESeq2 (Love et al., 2014), which calculated the log2 fold changes, Wald test p-values, and adjusted p-values (False Discovery Rate, FDR) by the Benjamini-Hochberg procedure. Significantly changed peaks were assessed with FDR<0.05.
Data quality	FastQC was used to check sequencing quality, mapping quality was ensured by uniquely mapping and duplication level, and peak quality was measured by peak enrichment at promoter and 5' UTR regions.<br>MYC EGFP rep1: 940 peaks were identified at FDR 5%. Of these 679 were show over five fold enrichment over input.<br>MYC EGFP rep2: 5396 peaks were identified at FDR 5%. Of these 2945 were show over five fold enrichment over input.<br>MYC OMOMYC rep1: 1 peaks were identified at FDR 5%. Of these 1 were show over five fold enrichment over input.<br>MYC OMOMYC rep2: 1 peaks were identified at FDR 5%. Of these 1 were show over five fold enrichment over input.<br>MYC SNF5 rep1: 137 peaks were identified at FDR 5%. Of these 102 were show over five fold enrichment over input.<br>MYC SNF5 rep2: 27 peaks were identified at FDR 5%. Of these 17 were show over five fold enrichment over input.
Software	Bowtie 2 - version 2.3.4.1, MACS2 - version 2.1.1.20160309, DiffBind - version 1.12.3, HOMER - version 4.10.

Flow Cytometry

Plots

Confirm that:

☒ The axis labels state the marker and fluorochrome used (e.g. CD4-FITC).

☒ The axis scales are clearly visible. Include numbers along axes only for bottom left plot of group (a 'group' is an analysis of identical markers).

☒ All plots are contour plots with outliers or pseudocolor plots.

☒ A numerical value for number of cells or percentage (with statistics) is provided.

Methodology

Sample preparation	Sample preparation is described in the Methods.
Instrument	Becton Dickinson LSRFortessa instrument
Software	BD FACSDDiva 8.0 software was used to analyze all flow cytometry data.
Cell population abundance	Cells were analyzed without sorting.
Gating strategy	For all flow cytometry experiments, on the SSC/FSC plots the dense population on both axes was used to select for single cells. For determining GFP-positive cells, an untransduced cell line control was measured for baseline fluorescence. All GFP-positive cells were gated against the untransduced control (see example in Supplementary data).

☒ Tick this box to confirm that a figure exemplifying the gating strategy is provided in the Supplementary Information.
